# Sclerotic prostate cancer bone metastasis: woven bone lesions with a twist

**DOI:** 10.1093/jbmrpl/ziae091

**Published:** 2024-07-23

**Authors:** Felipe Eltit, Qiong Wang, Naomi Jung, Sheryl Munshan, Dennis Xie, Samuel Xu, Doris Liang, Bita Mojtahedzadeh, Danmei Liu, Raphaële Charest-Morin, Eva Corey, Lawrence D True, Colm Morrissey, Rizhi Wang, Michael E Cox

**Affiliations:** Department of Urologic Sciences, University of British Columbia, Vancouver, BC V5Z 1M9, Canada; Vancouver Prostate Centre, Vancouver, BC V6H 3Z6, Canada; Department of Materials Engineering, University of British Columbia, Vancouver, BC V6T 1Z4, Canada; School of Biomedical Engineering, University of British Columbia, Vancouver, BC V6T 2B9, Canada; Department of Urologic Sciences, University of British Columbia, Vancouver, BC V5Z 1M9, Canada; Vancouver Prostate Centre, Vancouver, BC V6H 3Z6, Canada; Department of Urologic Sciences, University of British Columbia, Vancouver, BC V5Z 1M9, Canada; Vancouver Prostate Centre, Vancouver, BC V6H 3Z6, Canada; Department of Urologic Sciences, University of British Columbia, Vancouver, BC V5Z 1M9, Canada; Vancouver Prostate Centre, Vancouver, BC V6H 3Z6, Canada; Department of Urologic Sciences, University of British Columbia, Vancouver, BC V5Z 1M9, Canada; Vancouver Prostate Centre, Vancouver, BC V6H 3Z6, Canada; School of Biomedical Engineering, University of British Columbia, Vancouver, BC V6T 2B9, Canada; Centre for Aging SMART, Vancouver, BC V5Z 1M9, Canada; Department of Urologic Sciences, University of British Columbia, Vancouver, BC V5Z 1M9, Canada; Vancouver Prostate Centre, Vancouver, BC V6H 3Z6, Canada; Centre for Aging SMART, Vancouver, BC V5Z 1M9, Canada; Department of Orthopaedics, University of British Columbia, Vancouver, BC V5Z 1M9, Canada; International Collaboration on Repair Discoveries, Vancouver, BC V5Z 1M9, Canada; Department of Urology, University of Washington, Seattle, WA 98195, United States; Department of Urology, University of Washington, Seattle, WA 98195, United States; Department of Urology, University of Washington, Seattle, WA 98195, United States; Department of Materials Engineering, University of British Columbia, Vancouver, BC V6T 1Z4, Canada; School of Biomedical Engineering, University of British Columbia, Vancouver, BC V6T 2B9, Canada; Centre for Aging SMART, Vancouver, BC V5Z 1M9, Canada; Department of Urologic Sciences, University of British Columbia, Vancouver, BC V5Z 1M9, Canada; Vancouver Prostate Centre, Vancouver, BC V6H 3Z6, Canada; Centre for Aging SMART, Vancouver, BC V5Z 1M9, Canada

**Keywords:** bone QCT/microCT< analysis/quantitation of bone, tumor-induced bone disease< cancer, bone histomorphometry< analysis/quantitation of bone, indentation (nano/micro)< analysis/quantitation of bone, collagen< bone matrix

## Abstract

Bone metastases are the most severe and prevalent consequences of prostate cancer (PC), affecting more than 80% of patients with advanced PC. PCBMs generate pain, pathological fractures, and paralysis. As modern therapies increase survival, more patients are suffering from these catastrophic consequences. Radiographically, PCBMs are predominantly osteosclerotic, but the mechanisms of abnormal bone formation and how this pathological increase in bone density is related to fractures are unclear. In this study, we conducted a comprehensive analysis on a cohort of 76 cadaveric PCBM specimens and 12 cancer-free specimens as controls. We used micro-computed tomography to determine 3D organization and quantify bone characteristics, quantitative backscattering electron microscopy to characterize mineral content and details in bone structure, nanoindentation to determine mechanical properties, and histological and immunohistochemical analysis of bone structure and composition. We define 4 PCBM phenotypes: osteolytic, mixed lytic-sclerotic, and 2 subgroups of osteosclerotic lesions—those with residual trabeculae, and others without residual trabeculae. The osteosclerotic lesions are characterized by the presence of abnormal bone accumulated on trabeculae surfaces and within intertrabecular spaces. This abnormal bone is characterized by higher lacunae density, abnormal lacunae morphology, and irregular lacunae orientation. However, mineral content, hardness, and elastic modulus at micron-scale were indistinguishable between this irregular bone and residual trabeculae. The collagen matrix of this abnormal bone presents with irregular organization and a prominent collagen III composition. These characteristics suggest that osteosclerotic PCBMs initiate new bone deposition as woven bone; however, the lack of subsequent bone remodeling, absence of lamellar bone deposition on its surface, and presence of collagen III distinguish this pathologic matrix from conventional woven bone. Although the mineralized matrix retains normal bone hardness and stiffness properties, the lack of fibril anisotropy presents a compromised trabecular structure, which may have clinical implications.

## Graphical Abstract

**Graphical Abstract:**
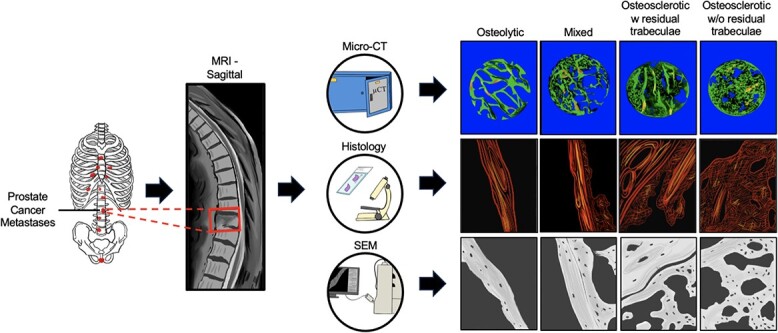

## Introduction

Prostate cancer (PC) is the most frequently diagnosed male cancer, with estimates of ~1.5 million new diagnoses every year.^1^ Although modern therapies have improved survival, about 20% of PC patients progress to an incurable metastatic state. Bone metastasis (BM) develops in >80% of these advanced PC patients^2^ and most frequently involves the trabecular bone of the axial skeleton (ribs, epiphyses of long bones, and the vertebral bodies).^3^^,^^4^ PCBM severely impair patients’ quality-of-life by causing intractable pain, bone weakness, and increased risk of fracture: significant morbidities and key indicators of mortality. The rich modern therapeutic spectrum is extending survival, resulting in patients living long enough for BMs to become increasingly relevant.^5^ It is therefore imperative that we better understand the biological mechanisms that cause BMs, and structural changes that they induce. This knowledge is vital for the rational development of effective treatments and palliative care options.

Because of their predominantly radio-dense presentation, PCBMs were histologically described as hyperosteoidosis^6^ or osteomalacia.^7^ Later analysis demonstrated that PCBMs exhibit a spectrum of pathologic changes from pronounced osteopenic to highly osteosclerotic phenotypes.^8^ Because of the lack of collagen matrix alignment in the osteosclerotic regions, the bone of PCBM has been described as “woven bone.” This term is used to describe poorly organized bone characteristic of fetal bone and the bone callus formed during fracture repair in adults; however, in both cases, woven bone is later replaced by lamellar bone.

Although informative, these are relatively low-resolution histologic descriptors, and only a handful of studies have ventured into high-resolution or 3D analysis of PCBMs. Some of the most informative studies are: a series of synchrotron-based high-resolution micro-computer tomography (μCT) scans from 2 patient samples,^9^^,^^10^which demonstrated alterations in BMD and trabecular morphology and structure, and recently, 2 high-resolution μCT scan studies reported similar structural changes in PCBMs obtained from 2 and 7 additional patients.^11^^,^^12^ These highly relevant descriptions are limited by sample size, and they require further validation to draw definitive conclusions.

Clinically, increased fracture risk in PC patients is linked to lower bone density due to hormone ablation therapy,^13^^,^^14^ while in male multiple myeloma patients, vertebral fractures are associated with trabecular thickening and sclerosis of 3 or more vertebrae, suggesting an association between fractures and osteosclerosis.^15^ Despite these critical effects in a large population, very few studies have studied the mechanical properties of bone affected by PCBM. To the best of our knowledge, a total of only about a dozen PCBM lesions have ever been structurally analyzed at high (micron) resolution or for their mechanical properties.^9-13^ Although these studies have made comparisons with bone lesions from other oncologic indications, they are limited by inclusion of no more than 2 PC specimens, and none have included healthy control samples against which to benchmark conclusions on the fracture risk of sclerotic lesions.

Here, we hypothesize that alterations in the structural organization of bone induced by PC can be identified that might indicate the cause of increased fracture risk. We aimed to perform a comprehensive investigation into the spectrum of alterations in composition, structure, and mechanical properties of vertebrae affected by PCBM. To achieve this goal, we use multiple high-resolution and 3D techniques to analyze a cohort of 76 cadaveric PCBM samples from 43 men and 12 samples from 4 healthy donors as control. The microstructure was analyzed by micro-computed tomography (μCT). High resolution morphology, calcium weight % (Ca wt%), properties of cellular lacunae were investigated through backscattered imaging in scanning electron microscopy. We explored the alignment of collagen in bone and complemented the analysis of the collagen composition of the extracellular matrix by immunohistochemistry. We observed a high variation in structure and mineral content among PCBMs that could be grouped in distinct phenotypes. Osteosclerotic PCBM are characterized by the presence of deposition of irregular mineralized matrix in the intertrabecular spaces, which present elevated lacunae density and size as well as poor collagen organization. This matrix also shows an increased content of collagen III, suggesting differences in the osteogenic process. Our findings provide the most comprehensive analysis of the spectrum of PCBM morphometric alterations ever undertaken and offer insights and foundational information into the characteristics of PCBM, while they have the potential to serve as a basis for investigating the mechanisms behind their formation and their clinical implications.

## Materials and methods

### Cadaveric vertebral metastasis of PC samples

All described procedures were approved by the institutional review boards at University of Washington (2341) and University of British Columbia (H21-02668). We obtained 76 cadaveric vertebral core samples from autopsies of 42 patients who died of metastatic PC, with mean survival of 8.1 yr from diagnosis (range 1.3–20.2). Further details regarding samples, and donor’s clinical and demographic conditions, are provided in Table S1. All patients provided written informed consent for a rapid autopsy to be performed ideally within 4 h of death, under the Prostate Cancer Donor Program at the University of Washington Medical Center.^16^ As control samples, we obtained 12 vertebrae from 4 age-matched male donors from the University of British Columbia Body Donation Program. The specimens were all trephine cores of 11 mm diameter, and between 1 and 3 cm length, obtained in an anterior–posterior direction from the ventral aspect of the vertebral bodies, and immediately stored in 70% ethanol until analysis.

### 3D μCT scanning of cadaveric PC bone

Sample processing and analysis for this study are summarized in Figure S1. The mineral structure of cadaveric vertebral samples was analyzed using a μCT scanner (Scanco Medical) with the following setting: 70 kVp, 114 mA, 8 W, 500 ms integration time. A total of 450 slices with an isotropic voxel size of 10 μm (total volume of 4.5 mm length, 1.1 cm diameter) were obtained on each specimen following the manufacturer’s standard protocol. To avoid considering the damage and debris created during trephination, a perimetric volume of 0.7 mm around the core circumferences was removed from each sample prior analysis. Areas with evidence of cortical bone, cartilage, or soft tissue were also excluded. We extracted the following bone parameters; trabecular bone volume/total volume (BV/TV), trabecular number (Tb.N), trabecular thickness (Tb.Th), trabecular separation (Tb.Sp), Connectivity Density (Conn-Dens). We used an automated segmentation algorithm (Image Processing Language, Version 5.08b, Scanco Medical) to determine BMD (mg HA/cm^3^).

We performed an initial qualitative assessment based on 3 30-μm thick CT-slices (3 voxels in depth) on each sample: one in the boundary between the top ¼ and the following ¼, one in the middle of the sample, and one in the boundary between the 3rd ¼ and the bottom ¼. We then assessed the resulting tomograms for the presence, and distribution, of trabeculae to define if the samples were osteolytic, mixed, or osteosclerotic PCBMs as described in the results section. PCBM have complex structures exhibiting areas of osteolysis, as well as osteosclerosis dominated by a disorganized matrix in the presence or absence of residual lamellar trabeculae). Statistical analysis was performed using the PRISM 10 (Graphpad) to perform one-way ANOVA, and unpaired *t* test followed by Tukey post hoc for multiple comparisons correction.

### Quantitative backscattered electron-scanning electron microscopy analysis of cadaveric PC bone specimens

After μCT scanning, 12 cadaveric specimens of PCBM representing 4 qualitatively defined morphological groups were cut transversally with a water-cooled diamond saw (IsoMet 4000) into 2 sections corresponding to 1/3 and 2/3 of the total length of the sample. The smaller bone section of each sample was demineralized and used for histology (Section Histology and immunohistochemistry), while the larger ones were immersed in a sequence of acetone (70%, 90%, and 100% ×2) for dehydration (2 d in each concentration). The dehydrated bone sections were then infiltrated with 50%, 80%, and 100% ×2 embedding medium (Spurr low viscosity kit, PELCO) in acetone for 3 d in each concentration under vacuum. After infiltration, bone sections were embedded together with Carbon (Spec-Pure Carbon Rod, TED PELLA) and Aluminum (99.99% Al, TED PELLA) standards for quantitative backscattered electron (BSE) analysis. The transverse surface of the embedded bone sections underwent grinding using a series of decreasing grit carbide papers and subsequently polished using a 1 μm diamond suspension. Following this preparation, the surface was coated with carbon and subjected to SEM ( FEI Quanta 650) imaging in BSE mode. The qBSE images were captured at a working distance of 15 mm with a magnification of 200×, resulting in image dimensions of 636 μm × 457 μm and a resolution of 1536 pixels × 1326 pixels. We determined the mineral density of an equal number (5–10 per sample) of random areas of interest, representing distinct bone morphologies in each specimen, expressed as calcium weight percent (Ca wt %; peak value representing the most prevalent calcium concentration) as previously described.^17^ The results for each sample and the overall results for trabecular vs sclerotic areas were compared by using *t*-test for paired samples.

### Lacunae analysis

Based on the analysis of BSE images, we selected 10 areas of interest within each of the 12 samples, ensuring representation across various lesion types, including osteosclerotic, mixed, and osteolytic. Within each image (636 μm × 457 μm; 1536 pixels × 1326 pixels), we determined areas of lamellar bone and woven/disorganized bone (sclerotic lesions) based on the presence or absence of a lamellar structure. We extracted information concerning lacunae properties (density, lacunae dimensions, lacunae area relative to bone area, and lacunae orientation) using a custom MATLAB script through multiple iterations in the following steps: the qBSE-SEM image is imported into the program, converted to grayscale, cropped to remove the info bar, and a Gaussian filter is applied. The grayscale threshold (corresponding to 8 Ca wt %) was determined to create a mask of the “voids” present in the bone. Voids with an area between 2.5 μm^2^ and 500 μm^2^ are identified as lacunae and designated with a unique identifier number. The area, centroid, major axis length, minor axis length, ratio between axes, circularity, eccentricity, and orientation (compared to horizontal) was derived for each lacuna. We manually excluded cracks and artifacts. We then quantified the average lacunae size, total lacunae area, average lacunae size, total region of interest area, number of lacunae, lacunae density, and the lacunae area/bone area fraction for every image and compared between bone vs sclerotic bone by using *t*-test for paired samples.

### Histology and immunohistochemistry

Sections obtained from the cadaveric specimens were fixed in 10% buffered formalin for 24 h, rinsed in PBS)3 times for 1 h each, and decalcified in 10% formic acid for 5 d. The specimens were then dehydrated in an ethanol series (70%, 80%, 90%, 95%, 100%), cleared in xylene substitute, and paraffin embedded. Serial 5 μm thickness sections were prepared and mounted on microscope slides. One slide of each core serial section was stained with picrosirius red, toluidine blue, and Goldners’ trichrome, respectively. Immunohistochemistry was performed following the protocol specified in Supplemental Methods and Table S2. The observations were performed using an upright microscope (Zeiss Axiophot). Images were obtained with a cooled camera and processed and collected using ZEN software (Carl Zeiss Canada). Image processing was performed using Adobe Photoshop.

**Figure 1:**
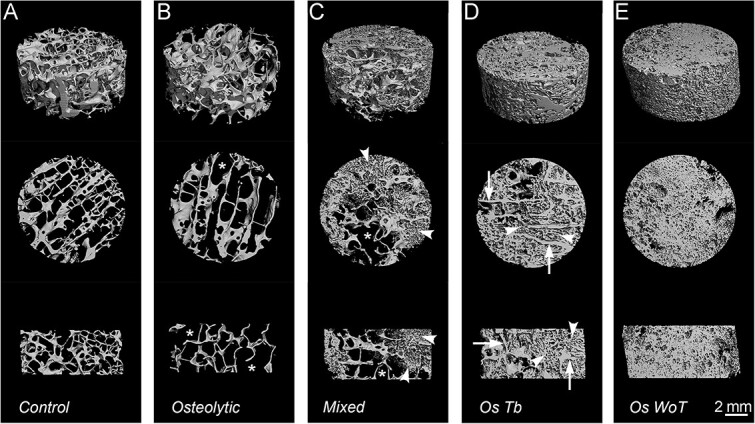
Renders of representative cadaveric vertebrae of every sub-type obtained from Micro-CT images. (A) Cadaveric age-matched control. (B) Osteolytic sample with large voids (asterisks) and thin-broken trabeculae (arrowhead). (C) Mixed PCBM sample, with areas of osteolysis (asterisks) characterized by large voids, and areas with increased bone component (arrowheads). (D) Osteoblastic PCBM sample, with residual trabeculae (arrows), which are thicker and characterized by deposition of mineral in the intertrabecular spaces (arrowheads). (E) Osteoblastic sample without residual trabeculae, showing a homogeneous matrix of irregular mineral deposition along the whole sample. Top views = 4.5 mm thick sections, middle and bottom sections = transverse view, and sagittal view of 1 mm thick sections. Renders were generated by superimposition of 6 μm sections.

## Results

### PCBM are variable, predominantly osteosclerotic, some osteolytic, and characterized by higher or lower trabecular number respectively

To evaluate the overall characteristics of the PCBMs, we performed quantitative μCT analysis of the whole core obtained from PC-involved vertebral samples (Table S3) and aged-matched controls (Table S4). We observed that of the 76 analyzed samples, 55 exhibited increased BV/TV compared to our age-matched control group (mean ± st. dev.). In contrast, only 9 samples exhibited lower BV/TV relative to the control group (Figure S2). Additionally, 55 of the samples have elevated Tb.N compared to controls, and only 3 have lower Tb.N. However, this higher Tb.N is not related to Tb.Th which, on average, were indistinguishable from the controls, but that exhibited a larger variation within and between samples. As expected, higher Tb.N is accompanied by lower Tb.Sp and higher Conn-Dens. We next assessed radiodensity of the mineral bone fraction of the specimens and observed that PCBM exhibited lower BMD than the control group. These results validate the consensus that PCBMs are predominantly osteoblastic lesions, with elevated volume of mineralized tissue, mostly due to a higher number of trabeculae, but no variation in their thickness. The smaller group of predominantly osteolytic lesions (~15%), with lower BV/TV, are characterized by having fewer trabeculae, rather than thinner trabeculae. It has been previously described that osteosclerotic bone is characterized by bone deposition on the surface of pre-existing trabeculae^16^^,^^18^ hence we hypothesize that this later result is due to the appearance of new fine trabecula in the osteosclerotic samples. We were unable to associate the data provided by the μCT analysis (BV/TV, Tb.N, TB.Th, Tb.Sp, Conn-Dens., and BMD) with clinical data variables (race, Gleason score, PSA at diagnosis, final PSA, Δ PSA, age at diagnosis, age at death, survival).

**Figure 2:**
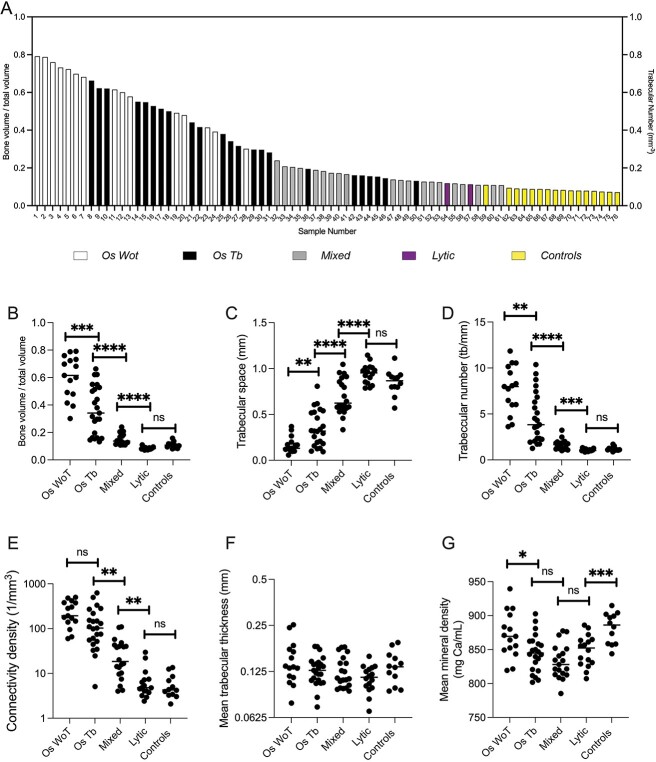
Quantitative analysis of samples based on lesions phenotype. (A) Waterfall plot of bone volume / total volume, of samples based on phenotypical presentation of micro-CT measurement. (B–G) Dot plots of quantification analysis of different sample phenotypes according to micro-CT evaluation. Statistical significance for each variable determined by ANOVA and comparisons between adjacent groups performed by unpaired *t*-test followed by Tuckey post-hoc. Os WoT = osteosclerotic without trabeculae, Os Tb = osteosclerotic with trabeculae. ^*^p < 0.05, ^*^^*^p < 0.01, ^*^^*^^*^p < 0.005, ^*^^*^^*^^*^p < 0.001, ns = not significant.

### PCBM have complex structures exhibiting areas of osteolysis, as well as osteosclerosis dominated by a disorganized matrix in the presence or absence of residual lamellar trabeculae

μCT analysis allows for a complete 3D reconstruction of the vertebral core sample. Combining a conventional spectrum of μCT measurements of trabecular bone with a qualitative analysis, we observed differences in mineralized matrix architecture in PCBM specimens that are generally consistent with those previously described in this cohort of specimens by histologic methods.^8^ Based on qualitative observation and using age-matched controls as baseline (Figure 1A), we define 4 distinct dysmorphic bone patterns in PCBM (Figure 1, color-coded images in Figure S3 display trabecular thickness in *z*-axis). Osteolytic samples (Lytic; *n* = 16) are those with at least 3 voids larger than 1 mm without trabeculae, and the presence of thinned or broken trabecula (Figure 1B). Mixed samples (Mixed; *n* = 20) exhibit both osteolytic and osteosclerotic areas (Figure 1C). Osteosclerotic with residual trabeculae (Os Tb; *n* = 23) are those in which we observed more than 5 trabeculae (each larger than 1 mm diameter) in each section accompanied by increased trabecular thickness and the presence of intertrabecular mineral material (Figure 1D). Osteosclerotic without residual trabeculae (Os WoT; *n* = 15) have less than 5 trabeculae per section and dominated by accumulation of irregular radio-dense material (Figure 1E).

Comparing the quantitative values provided by μCT with the qualitative classification of our morphological descriptions, we found that the μCT values are concordant with the differential structural pattern (Figure 2A and Table S5). When comparing specific parameters, we observed that the Os WoT specimens had an average BV/TV ratio that was significantly higher than that of the Os Tb specimens, which, not surprisingly, was higher than Mixed, Lytic, and control samples (Figure 2B). As expected, we observed the lowest Tb.Sp in the Os WoT compared with the Os Tb, which in turn was lower than mixed samples, and the highest values in the Lytic samples (Figure 2C). The Os WoT also exhibited a higher Tb.N than the Os Tb, which in turn is higher than that of the Mixed, which is higher than Lytic samples (Figure 2D). Although the Conn-Dens was indistinguishable between the osteosclerotic groups, their Conn-Dens was greater than that of the Mixed, and these exhibited a greater Conn-Dens value than Lytic and control samples (Figure 2E). We observed no differences in Tb.Th across the groups (Figure 2F). Surprisingly, we observed a higher BMD density in the Os WoT and control specimens compared to other groups, which in turn were indistinguishable (Figure 2G). Combined, these results suggest that different osteoblastic lesion phenotypes exist depending on the presence, or lack, of residual trabeculae that are evident by radiographical examination. The finding of higher mineral density in the Os WoT and the control group is suggestive of matrix alterations.

### Different PCBM phenotypes are present within a patient

To evaluate whether PCBM within a patient present with similar morphologic features, we analyzed samples from the 21 patients from whom we have 2 or 3 vertebral core samples using the previously described parameters. We first compared our qualitative phenotypic description for those samples and observed that 16 of these patients presented with variable phenotypes, while only 5 patients had 2 or more samples with similar phenotype (Table S6). To quantitatively evaluate the similarities, we compared the range of the BV/TV within a patient with the mean value of BV/TV for that patient. We observed that 8 of the 21 patients had a BV/TV range that varies less than 20% of the patient BV/TV average, while 13 have BV/TV variation that were greater than 20% of the patient’s average BV/TV ratio (Figure S4). This diversity of PCBM phenotypes and BV/TVs observed in the limited number of cases with more than one sample from a patient suggests that there is a considerable rate of intra-patient PCBM lesion heterogeneity.

### Irregular bone deposits on the surface of pre-existing trabeculae occupies the intertrabecular spaces in osteosclerotic PCBM

**Figure 3:**
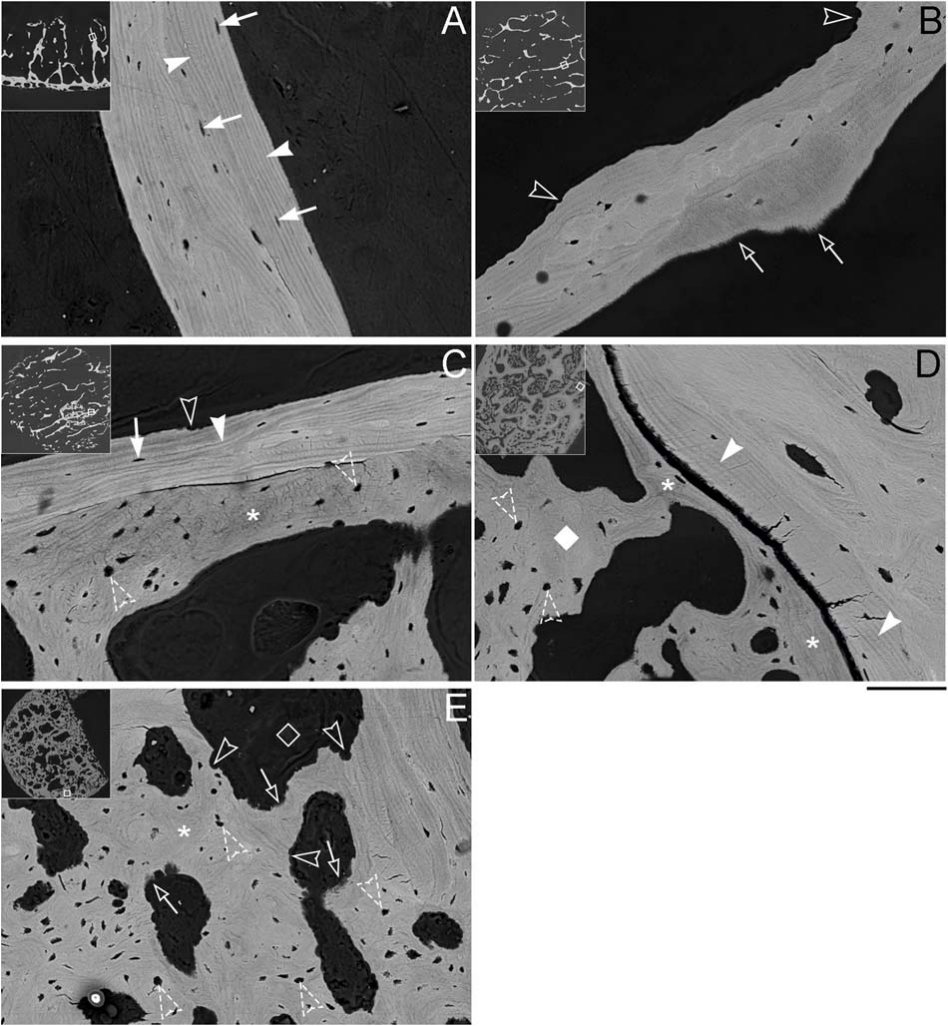
Backscattered electron microscopy observation of PCBM. (A) Aged-matched control sample, normal trabecular bone characterized by lamellar structure (arrowheads), and ovoid-shaped lacunae (arrows) oriented with its major axis following the orientation of lamellae. (B) Osteolytic sample, with evidence of Howship (osteoclasts) lacunae (open arrowheads), and irregular diffuse edge (open arrows) in the surface of trabeculae. (C) Mixed sample, where pre-existing trabeculae with regular lamellar structure (arrowhead) and osteocytes (arrow), in one side shows signs of bone resorption (open arrowhead), while in the other side presents abnormal bone deposition (asterisk), characterized by the absence of lamellar structure and irregular or round-shaped lacunae (dashed arrowheads). (D) Osteosclerotic sample with residual trabeculae, where the residual trabeculae can be identified by the presence of lamellar structure (arrowheads), and the deposition of irregular bone is evident on the surface of the pre-existing trabeculae (asterisks), and it extends toward the intertrabecular space (diamond). Noticeable is the gap or crack between the residual trabeculae and the irregular osteosclerotic bone. (E) Osteosclerotic sample without residual trabeculae, the space is filled with irregular osteosclerotic bone with no evidence of lamellar structure, abundant lacunae do not follow any specific orientation, and many of them are round-shaped (dashed arrowheads). The presence of numerous large voids is also noticeable (open diamonds). Howship lacunae (open arrowheads) and irregular edges (open arrows) may be indicative of active bone resorption. Bar = 100 μm. Insert is the low mag image of the sample analyzed (total width. = 1 cm), square in the insert shows area of the high magnification image. All SEM images obtained at 20 kV, WD = 15 mm.

To gain deeper insights into the microstructure of the PCBM vertebral specimens, we conducted high-resolution imaging analysis using SEM. We focused on 12 PCBM samples selected to represent the spectrum of PCBM presentations based on BV/TV and the qualitative observations previously described (Figure S5). Representative examples of the observed morphological features are shown in Figure 3.

Normal trabeculae from control samples (Figure 3A) displayed a characteristic lamellar structure, denoted by striations along the major axis of the trabecula (arrowheads), indicative of lamellar organization. Lacunae in these regions were elongated (arrows), with their major axis parallel to the lamellae. In osteolytic PCBM samples (Figure 3B), the trabeculae showed 2 characteristic features, one is the presence of Howship lacunae (open arrowheads), and the other one is the presence of irregular diffuse edges (open arrows). Mixed specimens (Figure 3C) showed evidence of both osteolytic activity (open arrowheads) and deposition of new bone (asterisk). This new bone is characterized by the absence of lamellar pattern and a larger and irregularly shaped lacunae (dashed open arrowheads). In Os Tb samples (Figure 3D), the presence of persisting trabeculae was discernible by the lamellar matrix (arrowheads) and the anisotropic lacunae (arrows). However, an abnormal, non-trabecular mineralized matrix was also evident on the surface of residual trabeculae (asterisks). This atypical, mineralized matrix is a hallmark of “sclerotic” regions in PCBM.^8^ It is characterized by the absence of lamellar lines and irregularly shaped lacunae (open arrows). This irregular sclerotic bone protruded from the trabecular surface into, and often occupied most of, the intertrabecular/medullary space (diamonds). Notably, distinct cracks were often observed between the sclerotic bone and the trabeculae in SEM imaging, possibly representing the boundary between trabeculae and sclerotic bone. In contrast, the Os WoT samples lacked regions of anisotropic trabeculae and were entirely composed of irregular matrix (Figure 3E), characterized by irregularly shaped lacunae and the presence of large irregular voids exceeding the size of normal lacunae (open diamonds). These observations suggest a differential composition of the osteosclerotic PCBM bone than trabecular bone, characterized by the absence of trabecular organization and irregular cellular distribution. These matrix characteristics are compatible with previous descriptions of woven bone in the pathologic architecture of PCBM.

### Unaltered mineral content is associated with normal microscale hardness and elastic modulus in osteosclerotic PC-woven bone

Given the distinct morphological characteristics of the sclerotic lesions, which exhibit differential BSE density compared to adjacent trabecular structures (Figure 3), coupled with our prior observation of varying mineral density in different PCBM types (Figure 2G), we postulated the presence of differing mineral content between irregular PC-associated sclerotic bone and residual trabeculae. To test this hypothesis, we conducted a qBSE-SEM analysis to assess the calcium content of the 12 specimens imaged with SEM. In each specimen, we measured at least 5 and up to 9 areas of both sclerotic and trabecular bone. Our findings revealed that the measured calcium content ranged from 21 to 25 Ca wt % across all specimens and regions (Table S7). Although some variations in Ca wt % were observed within specimens between sclerotic and trabecular bone, our analysis found no significant difference in calcium content between the sclerotic and trabecular bone across the examined regions of the 12 specimens (Figure S6). Although limited by sample number and area of analysis, these results suggests that there are no dramatic differences in the mineral content between osteosclerotic bone and trabecular bone.

The mechanical properties (hardness and elastic modulus) of bone are related to the clinical fractures and pain.^19^ To characterize the mechanical properties of the irregular PC-woven bone, we conducted nanoindentation tests at the micron level, following established protocols^20^ (Figure S6). For each specimen, we calculated the mean from a minimum of 10 measurements within trabecular and sclerotic regions, respectively, for hardness and elastic modulus. In the trabecular regions, the elastic modulus ranged from 7 to 16 GPa, with a mean value of 12.33 GPa, while in the sclerotic regions, the elastic modulus ranged from 7 to 13 GPa, with a mean value of 11.19 GPa (Figure S6B). Notably, the elastic modulus between the trabecular and sclerotic regions was indistinguishable (*p* value = 0.13). Furthermore, the elastic modulus values of these PCBM specimens are consistent with those previously reported for normal trabecular bone.^21^ Meanwhile, the measured hardness values between trabecular and sclerotic regions exhibited no discernible distinctions within or across the cancer-involved vertebral bone core specimens (Figure S6C). These values also aligned with those of normal trabecular bone. Given that our nanoindentation analysis included all the locations where we measured Ca wt %, we explored potential association between hardness, elastic modulus, and calcium mineral content at each measured site. We determined that there is no discernable association between calcium content and these mechanical properties (Figure S7). From these results, we conclude that despite noticeable morphological changes, PCBM sclerotic bone possesses mineralization, hardness, and elastic modulus that are indistinguishable from those of trabecular bone.

### Higher lacunae density, abnormal lacunae morphology, and orientation characterize osteosclerotic bone in PCBM

**Figure 4:**
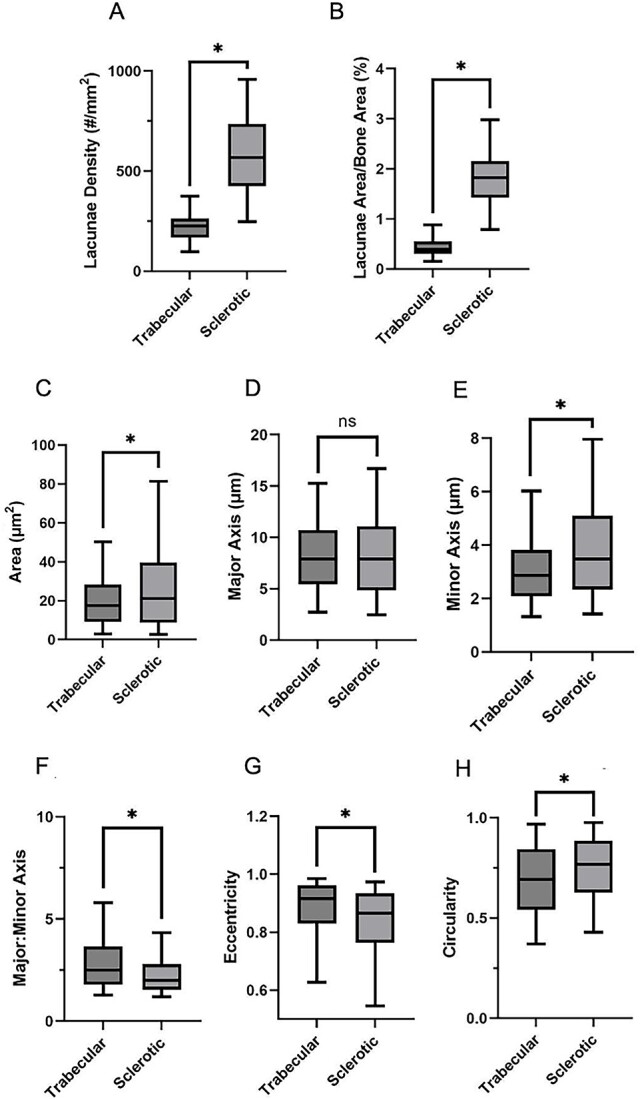
Characterization of PCBM lacunae morphology. Box plots of median ± 1 and 2 SD for measurements of cadaveric vertebral PCBM samples with regions of trabecular and sclerotic bone. (A) lacunae density, (B) bone area occupied by lacunae, (C) individual lacunae area, (D) major axis length. (E) minor axis length, (F) major-to-minor axis length ratio, (G) eccentricity, (H) circularity. ^*^p < 0.0001. *n* of analyzed lacunae in sclerotic areas = 3262; *n* of analyzed lacunae in trabecular areas = 1460.

**Figure 5:**
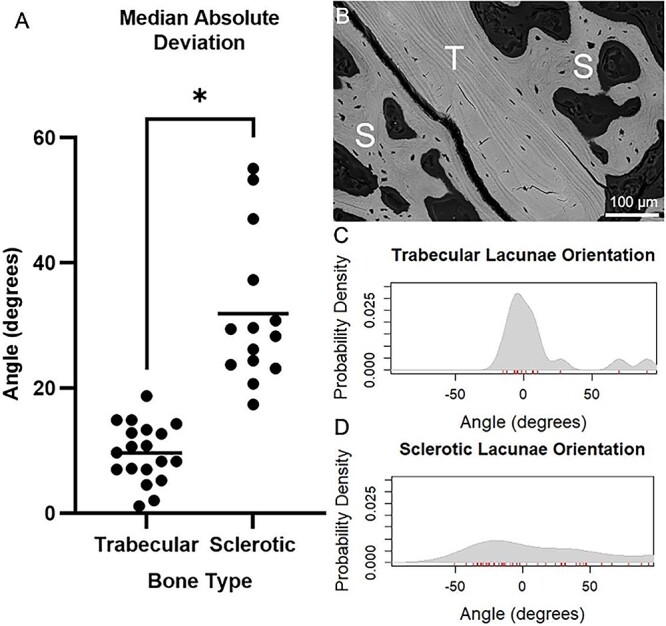
Lacunae orientation analysis in PCBM. (A) Median absolute deviation of the lacunae orientation for trabecular (*n* = 19) and sclerotic (*n* = 14) bone regions (^*^p < 0.0001. Total number of analyzed lacunae = 370 in trabecular bone, 718 in sclerotic bone). (B) Representative quantitative back scattered electron scanning electron microscopy image of PCBM vertebrae with a trabecular region (T) and a sclerotic region (S). (C) Probability density function for the lacunae orientation of trabecular bone in image B (*n* = 16 lacunae in this image). (D) Probability density function for the lacunae orientation of trabecular bone in image B and sclerotic bone (*n* = 50 lacunae in this image).

Lacunae house osteocytes, which are critical to bone modelling. Lacunae are also the sites that mechanically concentrate stress, favoring crack initiation and fracture development.^22^^,^^23^ In our investigation, we examined the lacunar properties within irregular PC-associated bone to ascertain how these lacunae differ from those within trabecular bone. Our 2D imaging analysis of SEM images of the 12 selected samples (Figure S5) unveiled a substantial (~4-fold) increase in both total lacunae density and area within the sclerotic regions compared to the trabecular regions (Figure 4A and B). Moreover, the lacunae within the sclerotic regions exhibited significantly larger sizes and distinctive “non-ovoid” shapes compared to regular lacunae (Figure 4C). This increase in size was primarily attributed to an elongation in the minor axis length rather than the major axis length (Figure 4D and E), implying that lacunae in the sclerotic lesion possess greater circularity and reduced eccentricity compared to those in trabecular regions (Figure 4F–H). Furthermore, we conducted an analysis of lacunae orientation based on our SEM images. The results revealed conspicuous differences in the distribution of angles representing the main axis of lacunae in osteosclerotic bone compared to the residual trabeculae (Figure 5). These observations suggest that lacunae in osteosclerotic bone do not exhibit alignment with a specific orientation, consistent with our earlier description of disordered collagen fibrils (Figure 3). Collectively, these findings highlight that sclerotic regions of PCBMs feature a larger and irregular distribution of lacunae and pores. This observation implies a higher number of stress accumulation points in osteosclerotic bone and potentially abnormal interaction between osteocytes within PCBM.

### Abnormal collagen deposition is accompanied by increased proteoglycan and phosphorylated glycoprotein content in irregular bone of PCBM

**Figure 6:**
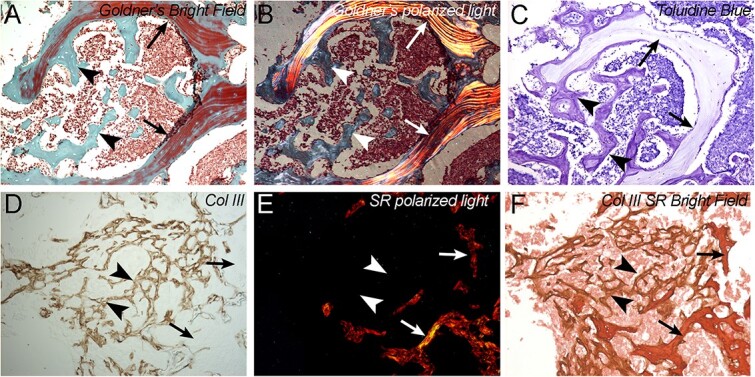
Histochemistry and immunohistochemistry of extracellular matrix proteins in PCBM. (A) Goldner’s trichrome staining of osteoblastic with residual trabeculae PCBM. Residual trabeculae are observed (arrows), along with disorganized osteosclerotic bone (arrowheads). (B) Polarized light imaging of sample slide in A. Birefringence corresponds to the staining of residual trabeculae but is not evident in osteosclerotic bone. (C) Toluidine blue staining of serial section of sample in A and B, showing stronger staining intensity in osteosclerotic bone as compared to residual trabeculae. (D) Anti-collagen III immunostaining of sample slide in E and F. Immunostaining is evident in osteosclerotic bone (arrowheads) but not in residual trabeculae (arrows). (E) Polarized light imaging of F, birefringence corresponds to trabeculae (bone), while osteosclerotic shows absence of birefringence. (F) Double stain Sirius red, anti-collagen III of osteoblastic with residual trabeculae PCBM. In bright field imaging, Sirius red stain trabeculae (arrows) and osteosclerotic bone (arrowheads). Images correspond to OSWT sample. Bar = 100 μm.

By using Sirius red and polarized light imaging, we observed a lower birefringence in the osteosclerotic bone compared with trabecular bone, suggesting poorer collagen alignment of PCBM (Supplemental methods, results, and Figure S8). Following the rationale that abnormal collagen deposition would be accompanied by changes in collagen composition, we went on to further histologically characterize these PCBM samples. Using Goldners trichrome as a method of differentiating immature (osteoid) and mature lamellar bone,^24^ we observed clear differential staining for trabecular bone and the sclerotic regions of PCBM specimens (Figure 6A). Taking advantage of the optical polarization properties of this staining method, we aligned strong birefringence in the trabecular regions defined in the bright field observations (Figure 6B). When using the cationic dye, toluidine blue, we observed that irregular PC-associated bone was more intensely stained than residual trabecular bone (Figure 6C), which suggests a higher negatively charged molecules content in osteosclerotic PC-associated bone. These methods provide independent validation of our classification of predominant PCBM lesion types and the delineation of residual trabeculae within the sclerotic lesions. Combined, these results define specific biochemical changes in the previous observation of irregular collagen structure and suggest the presence of other molecular alterations in the osteosclerotic PC bone.

Our observations of irregular collagen organization led us to evaluate the presence of collagen I, II, and III in the extracellular matrix (ECM) matrix of PCBM. By using Sirius red staining on the same IHC-stained slides, we could simultaneously observe collagen alignment and the presence of collagen III. We observed that PCBM exhibit areas that strongly stain with collagen III immunostaining forming an irregular network in the intertrabecular space (Figure 6D). While Sirius red staining shows absence of birefringence in these collagen III-rich areas, strong birefringence was observed in collagen III-negative trabeculae (Figure 6E). Collagen I is the most abundant structural protein of bone and is also observed in increased staining of non-birefringent bone deposited on the surface of residual trabecula, in the frank osteosclerotic areas (Figure S9). Collagen II staining resulted negative in all PCBM samples (Figure S9), suggesting the absence of cartilaginous composition on PCBM and demonstrating the presence of collagen III as a major component of sclerotic PCBM.

## Discussion

Because they cause tremendous, intractable pain and increase the risk of fractures, BMs are one of the most severe consequences of PC. Contradictorily, PCBM are osteosclerotic lesions with higher mineral content, but the mechanisms of pain and fracture, as well as the role of PC cells in this process, remain to be completely understood. In this study, we conducted a systematic evaluation of the structural and mechanical characteristics of PCBM, using what is by far, the largest cohort reported to date. We complemented our morphometric analysis with histological and immunohistochemical characterization of the PCBM extracellular matrix. Our findings revealed the prevalence of structural disparities in PCBM ranging from mixed osteolytic to profoundly osteodense, and, marked by the emergence of sclerotic PC-associated bone characterized by altered bone distribution, abnormal collagen organization, and higher porosity reminiscent of woven bone. Furthermore, our analysis indicates that these alterations in PCBM are not reflected on differences in the calcium content, or nano-scale hardness or elastic modulus. Instead, they appear to arise from a loss of 3D organization of the collagen matrix and increased porosity with more abundant and larger lacunae. In essence, our research sheds light on the complex interplay of structural and mechanical factors in PCBM, providing valuable insights into the mechanisms behind pain and fractures in PC patients.

X-ray imaging has historically been used to described 3 classes of PCBM lesions: those with elevated radiopacity, those that are radio lucid, and those that combine these 2 phenotypes described as mixed; however, these descriptions have been mostly based on patients’ X-ray or murine bone analysis after PC cells inoculation.^25^^,^^26^ High resolution tomography of PCBMs has been very limited. One study of vertebral PCBM from a single patient who died of PC, using a synchrotron-energized μCT scanner to analyze 15 7 mm isometric cubic sections of spine with a pixel size of 23.2 μm and a step size of 18.56 μm, reported higher bone surface and trabecular number in the PCBM compared to normal vertebrae, but no difference in trabecular thickness.^9^ The same group performed a similar analysis of vertebral lesions from 2 patients at 6 μm isometric voxel size, of 4 μm side cubes, to describe a higher BV/TV, bone surface area, trabecular number, trabecular connectivity, anisotropy, and fractal dimensions in PCBM.^10^

More recently, μCT scanning at an isometric voxel size of 10.5 μm was used to analyze 5 mm diameter cores obtained from cadaveric vertebra with metastasis from 2 men who died of PC, 3 patients who died of breast cancer and 3 from lung cancer.^11^ By complimenting this morphological analysis with mechanical testing and biochemical analysis, the PCBM cores were scored as sclerotic, lytic, or mixed, but for the statistical analysis, all samples were grouped according to imaging classification, thus no conclusion can be made from the specific cancer type. Finally, a μCΤ analysis of metastatic vertebrae from 2 patients, performed at a 24.5 μm isometric voxel size describes PCBM as osteoblastic, lytic, and/or mixed lesions.^13^ The results of analysis of specimens from these 7 patients to the best of our knowledge represent all of the micron-level resolution morphometrics of PCBMs using human samples. Being accrued across several distinct studies, using various acquisition and analysis methods, and all but one lacking comparison to control specimens limits the understanding of the spectrum of bone pathologies occurring in vertebral PCBMs.

By comparison, our observations are based on congruent analysis of the largest cohort of PCBM and non-PC controls described to date. Although our conclusions are consistent with the previous descriptions of sclerotic lesions (high BV/TV, Tb N, and Conn Dens, and no change in Tb Th), our cohort also reveals the extent and variation of morphometric patterns from sclerotic to lytic lesions and highlights 2 bone mineral patterns in osteoblastic lesions: those with and those without residual trabeculae. This differential pattern was previously observed from the histological observation of demineralized samples obtained from a distinct set from the same patient cohort^8^ in which osteoblastic lesions were described as “…consisting mostly of woven bone, with small amounts of osteoid and inclusions of native bone trabeculae appearing as lamellar bone… …In some cases an intact lamellar trabecular network remained, with woven bone around the edges of the normal bone.” Our results, combining tomographic analysis with high-resolution μCT scans, refine these conclusions by indicating that those with residual trabeculae have a residual trabeculae density indistinguishable from the density in sex- and age-matched controls, while those without residual trabeculae have compensated by expanding this “woven bone” feature to fully occupy the medullary space. A limitation of our work is that it is based on end-point analysis, leaving open the possibility that these distinct patterns involve either 2 distinct mechanisms: either that the absence or presence of a strong osteolytic activity drive development of lesions with or without residual trabeculae, or that these are part of a temporal continuum of modulated osteolytic and lamellar remodeling activity. Further confounding is our documentation intra-patient variability of PC vertebral lesions presenting as mixed/osteolytic and osteoblastic within proximal lumbar and lower thoracic vertebrae.

Because of its morphological characteristic, sclerotic PCBM lesions are generally described as “woven bone,”^27-29^ defined as a temporary mineralized bone matrix present during the initial synthesis of intramembranous lamellar bone^30^ in both embryonic development and fracture repair.^31^ Its relationship with trabecular bone is still not clear. It consists of a collagenous matrix composed of randomly oriented fibrils between 0.1 and 3 μm thickness, and calcium crystals which also show random orientation, generating a highly mineralized matrix but porous at the micron level.^32^ Woven bone is transient, and after its advantageous fast synthesis and mineralization on a fibrocartilaginous precursor, it is remodeled as lamellar bone in a process that starts by deposition of bone lamellae in the surface of woven bone.^31^^,^^33^ Woven bone is histologically characterized by the presence of a “meshwork of collagen bundles which bear no fixed relationship either to the lacunae or vascular spaces of the tissue.”^30^ Based on the extensive work of Dr Marotti, woven bone has been described as lacking lamellar structure and is characterized by having rounded lacunae within a fibrillar collagen I matrix^34^ with some differences in protein composition.^35^

Our observations of sclerotic PCBMs partially agree with these descriptions. We observed irregular matrix deposition, with poor collagen alignment and rounded lacunae, which in turn are not aligned with any structures. Conversely, we did not observe any evidence of cartilaginous matrix neither by histochemical staining nor by immunohistochemistry. Similarly, we did not observe evidence of lamellar or aligned collagen deposition on the surface of woven bone, and no resorption of this structure that could lead to a later stage of woven bone life cycle.^31^ Quite to the contrary, we find that matrix deposited on lamellar surface is abruptly non-birefringent, and based on cracking patters, not in union with the pre-existing lamellar matrix. Our observation of collagen III accumulation in sclerotic PCBM matrix is not traditionally described in woven bone. It is, however, described during the bone wound healing process,^36^ concurring with Dvorak’s description of cancer as “wounds that do not heal.”^37^

Collagen III is a fibrillar collagen commonly associated to collagen I in large fibers in tendon and skin, and combining with reticulin, forms reticular fibers that provide the structural scaffold to most internal organs. In bone, collagen III is involved in the formation of trabecular bone^38^ and forms a scaffold that precedes cartilage formation in endochondral osteogenesis.^39^ Its presence in PCBM matrix might represent an early stage of bone formation that never progress to a mature lamellar stage. Collagen III is reported to be essential for bone migration and invasion in breast cancer models.^40^ Collagen III has well-described roles in mesenchymal cells, in the progression and survival of epithelial cancers, and the modification of its extracellular matrix by cancer-associated fibroblasts that produce an altered ECM that promotes cancer cell migration and survival.^41^ Collagen III is, therefore, important in forming a scaffold for tumors that promotes cell adhesion, survival, and migration, as well as promoting neovascularization. In bone, the mesenchymal cells are osteoblasts and osteocytes. Although they have been implicated in metastatic progression,^42^ the precise mechanism(s) remain to be described. These analyses of altered bone matrix characteristics in PCBMs provide a new framework for characterizing cancer-associated osteoblasts and osteocytes in appropriately collected PCBM biobank specimens.

Bone hierarchical structure is critical for its mechanical properties. The organization of collagen fibers and lamellar structure allows elastic and inelastic deformation, which enhance the material toughness.^43^ Additionally, lamellae are instrumental in resisting crack initiation and propagation.^22^ These lamellar structures counteract the “weakness” that results from the stress accumulation in the pores of biomineralized composite materials.^44^ Furthermore, the small canaliculi are initiation points for microcrack that allows inelastic deformation, increasing bone toughness, while at the same time a dense collagen network prevents crack propagation and bone fracture.^45^ The structural alterations that we observed in PCBM, theoretically, would lead to increased weakness. Due to the enhanced porosity caused by larger lacunae number, size, and irregular shape, the chances of crack initiation would increase under tensile and compressive forces, while the absence of trabecular structure would not prevent crack propagation. Interfaces between 2 different materials are also stress accumulation areas, as we observed in our samples, these material differences may also be a starting point of cracks. With minimum or non-bonding mechanisms between irregular PCBM-associated bone and pre-existing trabeculae, these cracks may easily propagate through the interface, further increasing the risk of fracture. However, this latest hypothesis still lacks experimental demonstration.

Bone is a common site of metastasis for a variety of cancer types such as breast, renal cell carcinoma, lung, multiple myeloma, and prostate. It is accepted that complex interactions with the bone marrow environment determine survival and proliferation of cancer cells as well as bone turnover.^46^^,^^47^ Most of these interactions lead to osteolytic lesions by activating the receptor activator of NF-κB ligand (RANK-L)/RANK/ axis.^8^^,^^47^ Nevertheless, most PCBM are osteoblastic, which suggests a different interaction of PC cells with the bone niche. As a result of in vitro models, it is thought that patients with androgen-dependent PC are more prone to develop osteoblastic lesions, while those with androgen-independent PC develop osteolytic lesions.^48^^,^^49^ However, our results showing that a single patient can simultaneously develop osteolytic and osteosclerotic lesions provide arguments against this hypothesis, and more work needs to be done to understand the mechanisms of this variability.

As main limitations of our work, we acknowledge that the sample number for the analysis of SEM-qBSE is still reduced, and a larger cohort needs to be analyzed. Similarly, the analysis of lacunae is limited to a 2D image of the surface of SEM images, and thus 3D analysis is needed to confirm those findings. In the μCT analysis, we report the largest sample number ever reported in this type of analysis; however, the area that we could analyze for each lesion has a size limitation given by the sample obtention method. Our histological observations were performed on a limited number of samples and the slides were limited to the volume of lesion that we could observe in parallel to SEM observations.

The alteration of collagenous and non-collagenous ECM of irregular PC-associated bone that we describe in this article is novel in human tissues, but some interesting observations have been explored previously in animal models. High performance liquid chromatography and Raman spectroscopy analysis of osteoblastic murine bone lesions generated after HELA cell injection exhibited lower collagen crosslinking and mineral crystallinity, suggesting an alteration in the mineralization process of bone.^44^ Concordantly, Raman spectroscopy analysis of LNCaP C4-2B inter-tibial osteoblastic lesions was characterized by the presence of collagen with lower mineral ratio than normal bone, and the calcium component showed lower crystallinity and higher carbonate substitution compared to the non-affected contralateral tibiae.^30^ A similar analysis of sclerotic lesions caused by inoculation of PCa-2b cells revealed higher bone volume, bone volume fraction, BMC, and mineral density compared to control.^32^ By quantitative polarized microscopy, the authors determined reduced collagen alignment in the newly formed bone, and by using X-ray diffraction they determined lower crystal alignment in the PC-induced bone. Our results in human samples find concordance with some of these results, in terms of collagen arrangement, while further analysis in proteins and mineral phase are needed to establish definitive conclusions.

## Supplementary Material

Eltit_et_al_JBMR_Plus_Supplement_ziae091

## Data Availability

The data underlying this article are available on request to the corresponding author.
