# Supplementary material for: Sclerotic prostate cancer bone metastasis: woven bone lesions with a twist
Source: JBMR Plus. 2024 Jul 23;8(10):ziae091. doi: 10.1093/jbmrpl/ziae091 (PMC11365963; doi:10.1093/jbmrpl/ziae091)
Supplement: Eltit_et_al_JBMR_Plus_Supplement_ziae091 [file eltit_et_al_jbmr_plus_supplement_ziae091.docx]

**Supplemental Materials**

**Sclerotic prostate cancer bone metastasis: woven bone lesions with a twist.**

**Felipe Eltit*^1,2^, Qiong Wang*^3,4^,** Naomi Jung^1,2^, Sheryl Munshan^1,2^, Dennis Xie^1,2^, Samuel Xu^1,2^, Doris Liang^3,4^, Bita Mojtahedzadeh^1,2^, Danmei Liu^5^, Raphaële Charest-Morin ^7,8^, Eva Corey^6^, Lawrence D True^6^, Colm Morrissey^6^**,** Rizhi Wang^3,4,5^, Michael E. Cox^1,2,5^

Corresponding author: Michael Edward Cox: 2660 Oak Street, Vancouver, BC V6H 3Z6. E-mail: [mcox@prostatecentre.c](about:blank)om . Phone: (1) 604-875-4818

^1^ Department of Urologic Sciences, University of British Columbia, Vancouver, Canada.

^2^ Vancouver Prostate Centre, Vancouver, Canada.

^3^ Department of Materials Engineering, University of British Columbia, Vancouver, Canada.

^4^ School of Biomedical Engineering, University of British Columbia, Vancouver, Canada.

^5^ Centre for Aging SMART, Vancouver, Canada.

1. Department of Urology, University of Washington, Seattle, USA.
2. Department of Orthopaedics, University of British Columbia, Vancouver, Canada.
3. International Collaboration on Repair Discoveries, Vancouver, Canada.

* FEG and QW contributed equally to this work

Running title: Structure of prostate cancer bone metastasis

**SUPPLEMENTAL METHODS**

**Nanoindentation**

We employed a nanoindentation system (Nano Indenter XP System, MTS Nano Instruments, Oak Ridge, TN, USA) featuring a Berkovich tip to assess the elastic modulus and hardness of the embedded bone samples. Prior to the measurements, the area function in the nanoindentation system was calibrated using the Young’s modulus of fused silica (72 Gpa), and the indenter tip was calibrated relative to the microscope, before initiating the indentation process. Nanoindentation measurements were conducted on the polished embedded bone sections in areas that were previously analyzed with qBSE (2.3), ensuring a controlled penetration depth of 1.0 µm. The loading strain rate was set at 0.05 s^-1^, with a holding time at the peak load of 10 seconds. Subsequently, the tip was unloaded to 90% of its peak load. The elastic modulus and hardness values were derived from the load versus displacement curve utilizing the Oliver and Pharr method ^(18)^. The comparison of Young’s modulus and hardness between trabecular and sclerotic areas was performed using a t-test for paired samples.

**Immunohistochemistry**

For immunohistochemical analysis, we performed antigen retrieval on mounted sections with 2% pepsin in 0.1 N HCl for 20 minutes at 37 °C, followed by endogenous peroxide quenching using 3% H_2_O_2_ for 15 minutes. For non-specific protein blocking we used 3% bovine serum albumin (BSA) in PBS for 20 minutes. We incubated the samples with primary antibodies (list of antibodies in Table S2) diluted in 3% BSA in PBS overnight at 4 °C in humidified chamber. After washing with PBS, we incubated the samples in horse radish peroxidase (HRP)-conjugated secondary antibodies (Table S2) for one hour at room temperature. We revealed the immune detection using a commercial detection kit (VECTOR) following manufacturer instructions.

**Sirius red staining and collagen alignment quantification**

We used Picrosirius red staining to quantify birefringence as an indirect method of measuring collagen alignment ^(19)^. Images including ~30% of the histological sections were taken under bright field and polarized light. Pictures with a field of view of 6 x 6 mm were obtained using low magnification in the centre of the tissue section. We used ImageJ software to quantify the bone area, by measuring the percentage covered by bone in each image using the red channel in pictures taken bright field. The birefringence was calculated by measuring the area detected by the green channel in polarized light images. The comparison between groups was performed by using one-way ANOVA.

**SUPPLEMENTAL RESULTS**

**3.7 Irregular collagen alignment in osteosclerotic bone characterize PCBM**

Based on SEM imaging, we qualitatively observed a lack of lamellar structure in the irregular PC-associated bone (Fig. 4). Lamellae are critical structures in hierarchical organization of bone. They are composed of highly aligned “anisotropic” collagen fibres, which in trabecular bone are organized in lamellar packets directionally oriented following Wolff’s law ^(26)^, and it is the way mineralized collagen fibers provide bone strength ^(27,28)^. The optical polarization properties of Sirius red allow for visualization of changes in fibrillar collagen birefringence that are related to changes in collagen isotropy ^(29)^. We observed strong polarization of collagen fibrils in trabecular regions, and a reduced birefringence in sclerotic regions of PCBM specimens defined as being osteosclerotic with evidence of residual trabeculae (Fig. 8A-F). Consistent with μCT measurements of the entire cores, Lytic samples showed lower BV/TV than Os Tb, or Os WoT specimens, but here in thin sections, BV/TV of the two osteosclerotic groups was indistinguishable (Fig. 8G). When comparing the birefringent area in these samples, we found that the highest proportion of birefringent area was in the PCBM samples defined as osteolytic (Fig. 8H). The birefringent area (as part of the total bone area) of those defined as Os Tb was reduced by 20%, while that of those defined as Os WoT was half that of the Lytic specimens.

**SUPPLEMENTAL TABLES**

**Table S1: Demographics and clinical data of patients.**

| **Patient** | **Vertebrae** | **Sample No** | **Race** | **Gleason primary** | **PSA at Ddx** | **Final Serum PSA (ng/mL)** | **Δ PSA** | **Age at Diagnosis** | **Age @ death (years)** | **survival from dx (years)** |
| --- | --- | --- | --- | --- | --- | --- | --- | --- | --- | --- |
| 1 | L1 | 42 | C | 4+3 | 10.5 | 21.16 | 37.58 | 48.08 | 52.15 | 4.07 |
|  | L2 | 72 |  |  |  |  |  |  |  |  |
|  | L3 | 73 |  |  |  |  |  |  |  |  |
| 2 | L2 | 45 | C | 4+5 | 2.6 | 0.5 | 50.13 | 52.73 | 58.06 | 5.33 |
|  | L4 | 43 |  |  |  |  |  |  |  |  |
|  | L5 | 60 |  |  |  |  |  |  |  |  |
| 3 | L2 | 16 | M | 5+4 | 105 | 620.5 | -47.16 | 57.84 | 59.94 | 2.09 |
| 4 | L1 | 47 | C | 4+4 | 6.2 | 1 | 50.34 | 56.54 | 60.16 | 3.62 |
|  | L2 | 50 |  |  |  |  |  |  |  |  |
|  | L3 | 56 |  |  |  |  |  |  |  |  |
| 5 | L1 | 21 | C | 5+4 | 76 | 254.49 | -16.30 | 59.70 | 61.00 | 1.30 |
|  | T11 | 28 |  |  |  |  |  |  |  |  |
| 6 | L1 | 43 | C | 4+5 | 6 | 0.15 | 53.22 | 59.22 | 61.60 | 2.38 |
|  | L2 | 58 |  |  |  |  |  |  |  |  |
| 7 | L2 | 62 | C | 4+3 | 20.6 | 967.48 | 30.08 | 50.68 | 62.22 | 11.54 |
|  | L4 | 66 |  |  |  |  |  |  |  |  |
|  | L1 | 74 |  |  |  |  |  |  |  |  |
| 8 | L5 | 13 | C | 4+5 | 15.8 | 2534.86 | 43.37 | 59.17 | 63.44 | 4.27 |
| 9 | T11 | 23 | C | 4+3 | 64.6 | 3869 | -5.02 | 59.58 | 64.04 | 4.46 |
| 10 | L4 | 10 | C | 3+5 | 8.6 | 106.1 | 54.52 | 63.12 | 64.71 | 1.59 |
|  | L3 | 15 |  |  |  |  |  |  |  |  |
|  | L5 | 30 |  |  |  |  |  |  |  |  |
| 11 | L5 | 6 | C | 4+5 | 1000.1 | 767.6 | -938.11 | 61.99 | 65.19 | 3.19 |
|  | L4 | 11 |  |  |  |  |  |  |  |  |
|  | L2 | 17 |  |  |  |  |  |  |  |  |
| 12 | L2 | 4 | M | 4+3 | 4 | 380.95 | 57.56 | 61.56 | 66.43 | 4.87 |
|  | L1 | 14 |  |  |  |  |  |  |  |  |
|  | L3 | 20 |  |  |  |  |  |  |  |  |
| 13 | L2 | 52 | C | 4+5 | 1.7 | 58.74 | 61.68 | 63.38 | 67.20 | 3.82 |
| 14 | L2 | 68 | C | 4+5 | 0 | 0.72 | 58.49 | 58.49 | 67.28 | 8.79 |
| 15 | L3 | 63 | C | 4+5 | 14 | 454 | 50.97 | 64.97 | 67.49 | 2.52 |
|  | L1 | 64 |  |  |  |  |  |  |  |  |
| 16 | L1 | 54 | M | 4+4 | 226 | 328.3 | -159.91 | 66.09 | 69.16 | 3.07 |
| 17 | L5 | 69 | C | 3+4 | 13 | 104.4 | 49.67 | 62.67 | 70.12 | 7.45 |
| 18 | L4 | 49 | C | 4+4 | 20 | 90.94 | 43.49 | 63.49 | 71.21 | 7.72 |
| 19 | T12 | 26 | C | 7 | 33.9 | 105.6 | 28.17 | 62.07 | 71.64 | 9.56 |
| 20 | L5 | 36 | C | 9 | 0 | 119.7 | 61.50 | 61.50 | 72.39 | 10.89 |
|  | L4 | 39 |  |  |  |  |  |  |  |  |
|  | L1 | 57 |  |  |  |  |  |  |  |  |
| 21 | L5 | 33 | C | 4+3 | 12 | 2511.73 | 51.66 | 63.66 | 72.65 | 8.99 |
| 22 | L4 | 41 | C | 3+4 | 5.4 | 2816 | 54.25 | 59.65 | 72.88 | 13.23 |
| 23 | L5 | 38 | M | 4+4 | 54.8 | 290.1 | 15.29 | 70.09 | 72.93 | 2.84 |
|  | L2 | 61 |  |  |  |  |  |  |  |  |
|  | L4 | 71 |  |  |  |  |  |  |  |  |
| 24 | L3 | 29 | C | 7 | 42 | 3.3 | 19.68 | 61.68 | 73.14 | 11.46 |
|  | L5 | 51 |  |  |  |  |  |  |  |  |
| 25 | L2 | 22 | C | 8 | 41 | 359 | 21.84 | 62.84 | 75.43 | 12.59 |
|  | L4 | 65 |  |  |  |  |  |  |  |  |
|  | L3 | 70 |  |  |  |  |  |  |  |  |
| 26 | L4 | 5 | C | 3+4 | 13.4 | 547 | 56.96 | 70.36 | 76.02 | 5.66 |
|  | L2 | 7 |  |  |  |  |  |  |  |  |
|  | L1 | 24 |  |  |  |  |  |  |  |  |
| 27 | L1 | 25 | C | 4+3 | 12 | 3700 | 47.84 | 59.84 | 76.59 | 16.75 |
|  | L4 | 3 |  |  |  |  |  |  |  |  |
|  | L3 | 8 |  |  |  |  |  |  |  |  |
| 28 | L1 | 2 | C | 3+3 | 10 | 73.1 | 55.15 | 65.15 | 76.84 | 11.69 |
|  | L4 | 27 |  |  |  |  |  |  |  |  |
| 29 | L4 | 44 | M | 5+5 | 12 | 762.3 | 60.57 | 72.57 | 77.13 | 4.56 |
|  | L3 | 48 |  |  |  |  |  |  |  |  |
| 30 | L1 | 34 | C | 4+4 | 10.2 | 1250.4 | 53.02 | 63.22 | 79.52 | 16.31 |
| 30 | L5 | 67 | C | 4+5 | 49.1 | 5.63 | 29.26 | 78.36 | 80.83 | 2.48 |
| 32 | T11 | 35 | C | 5+5 | 27.2 | 16.6 | 48.24 | 75.44 | 81.02 | 5.58 |
| 33 | L3 | 19 | M | 3+3 | 6.2 | 2295 | 63.73 | 69.93 | 81.71 | 11.78 |
| 34 | L3 | 45 | C | ? | ? | 63.23 | 68.63 | 68.63 | 82.53 | 13.89 |
| 35 | L5 | 55 | C | 2+3 | 0 | 30.7 | 68.49 | 68.49 | 82.98 | 14.49 |
| 36 | L2 | 40 | C | 3+4 | ? | 114.83 | 72.98 | 72.98 | 83.47 | 10.49 |
| 37 | L1 | 31 | C | 3+4 | 43 | 240.65 | 31.62 | 74.62 | 83.70 | 9.08 |
| 38 | L1 | 59 | C | 4+3 | 12.4 | 721.9 | 61.37 | 73.77 | 85.67 | 11.90 |
|  | L3 | 75 |  |  |  |  |  |  |  |  |
| 39 | T12 | 32 | M | 4+3 | 10 | 1508.4 | 64.30 | 74.30 | 86.06 | 11.76 |
| 40 | L4 | 9 | C | ? | 0 | 413.2 | 65.98 | 65.98 | 86.23 | 20.25 |
|  | L3 | 12 |  |  |  |  |  |  |  |  |
|  | L5 | 76 |  |  |  |  |  |  |  |  |
| 41 | L4 | 18 | C | 3+5 | 11.1 | 349.05 | 61.39 | 72.49 | 90+ | 20.21 |
| 42 | L4 | 25 | C | ? | ? | 1455.9 | ? | 78.65 | 90+ | 11.18 |
|  | L2 | 37 |  |  |  |  |  |  |  |  |

Sample no= sample number assigned according to the sample ranking in the bone volume / total volume values from micro-CT scans (Fig 1A in main manuscript). Race = Caucasian (C) or Mixed (M). Gleason score corresponds to pathology evaluation after proctectomy. PSA at Dx = value for serum prostate specific antigen (PSA) at PC Diagnosis. Final Serum PSA = last value of PSA obtained prior to death. D PSA = PSA at Dx - Fina Serum PSA.

**Table S2: Antibodies used in IHC.**

| **Primary antibodies** | | | | |
| --- | --- | --- | --- | --- |
| **Antigen** | **Host species** | **Brand** | **Cat No** | **Dilution** |
| Col 3 | Mouse | Abcam | 6310 | 1/100 |
| Col 1 | Rabbit | Bioss | BS-0578R | 1/100 |
| Col2 | Mouse | Millipore | MAB8887 | 1/100 |
| **Secondary antibodies - HRP conjugated** | | | |  |
| Mouse Igg | Goat | Abcam | 205719 | 1/1000 |
| Rabbit Igg | Goat | Abcam | 205718 | 1/1000 |

**Table S3: micro-CT mean values in PCBM.**

| **Sample No** | **Patient** | **BV/TV** | **Tb.Sp** | **Tb.N** | **Conn-Dens.** | **BMD** | **Tb.Th** |
| --- | --- | --- | --- | --- | --- | --- | --- |
| 42 | 1 | 0.161 | 0.555 | 1.9 | 32.6 | 801.7 | 0.113 |
| 72 | 1 | 0.079 | 0.790 | 1.3 | 22.1 | 807.1 | 0.069 |
| 73 | 1 | 0.078 | 0.902 | 1.1 | 7.4 | 849.0 | 0.083 |
| 45 | 2 | 0.154 | 0.524 | 2.0 | 75.4 | 811.6 | 0.105 |
| 53 | 2 | 0.124 | 0.569 | 1.8 | 39.3 | 814.9 | 0.097 |
| 60 | 2 | 0.109 | 0.579 | 1.8 | 20.2 | 821.2 | 0.097 |
| 16 | 3 | 0.528 | 0.099 | 9.4 | 627.1 | 842.5 | 0.105 |
| 47 | 4 | 0.138 | 1.046 | 1.0 | 4.0 | 840.6 | 0.180 |
| 50 | 4 | 0.132 | 0.807 | 1.3 | 5.1 | 850.0 | 0.139 |
| 56 | 4 | 0.114 | 0.855 | 1.2 | 5.2 | 838.5 | 0.125 |
| 21 | 5 | 0.441 | 0.207 | 5.7 | 153.7 | 839.6 | 0.127 |
| 28 | 5 | 0.301 | 0.330 | 3.6 | 170.5 | 849.2 | 0.132 |
| 43 | 6 | 0.160 | 0.605 | 1.7 | 24.6 | 818.6 | 0.110 |
| 58 | 6 | 0.110 | 0.600 | 1.7 | 13.6 | 821.5 | 0.097 |
| 62 | 7 | 0.095 | 0.853 | 1.2 | 4.8 | 830.8 | 0.125 |
| 66 | 7 | 0.088 | 0.964 | 1.0 | 3.8 | 835.0 | 0.132 |
| 74 | 7 | 0.074 | 0.981 | 1.0 | 4.8 | 816.1 | 0.101 |
| 13 | 8 | 0.578 | 0.170 | 8.0 | 378.5 | 855.6 | 0.117 |
| 23 | 9 | 0.415 | 0.199 | 6.0 | 374.4 | 852.4 | 0.078 |
| 10 | 10 | 0.621 | 0.182 | 7.4 | 147.5 | 871.7 | 0.135 |
| 15 | 10 | 0.548 | 0.294 | 5.1 | 130.1 | 880.9 | 0.143 |
| 30 | 10 | 0.296 | 0.540 | 2.2 | 60.7 | 858.2 | 0.139 |
| 6 | 11 | 0.698 | 0.099 | 10.6 | 500.4 | 883.2 | 0.136 |
| 11 | 11 | 0.616 | 0.167 | 6.0 | 95.0 | 879.9 | 0.185 |
| 17 | 11 | 0.513 | 0.195 | 6.3 | 143.8 | 843.0 | 0.153 |
| 4 | 12 | 0.732 | 0.119 | 8.3 | 139.4 | 870.9 | 0.168 |
| 14 | 12 | 0.551 | 0.130 | 8.1 | 270.0 | 848.7 | 0.120 |
| 20 | 12 | 0.479 | 0.131 | 8.1 | 282.6 | 819.2 | 0.101 |
| 52 | 13 | 0.127 | 0.700 | 1.5 | 14.2 | 833.5 | 0.112 |
| 68 | 14 | 0.084 | 1.010 | 1.0 | 10.8 | 828.7 | 0.095 |
| 63 | 15 | 0.091 | 0.787 | 1.3 | 29.6 | 825.3 | 0.104 |
| 64 | 15 | 0.090 | 1.104 | 0.9 | 4.1 | 863.4 | 0.135 |
| 54 | 16 | 0.119 | 0.623 | 1.6 | 16.4 | 875.0 | 0.103 |
| 69 | 17 | 0.083 | 1.145 | 0.9 | 2.4 | 855.5 | 0.117 |
| 49 | 18 | 0.133 | 0.529 | 2.0 | 57.3 | 819.8 | 0.095 |
| 26 | 19 | 0.341 | 0.314 | 3.7 | 98.6 | 862.6 | 0.140 |
| 36 | 20 | 0.195 | 0.338 | 3.0 | 51.6 | 841.6 | 0.120 |
| 39 | 20 | 0.173 | 0.526 | 2.0 | 38.2 | 806.5 | 0.108 |
| 57 | 20 | 0.113 | 0.782 | 1.3 | 7.4 | 844.8 | 0.102 |
| 33 | 21 | 0.208 | 0.583 | 1.9 | 16.7 | 852.0 | 0.142 |
| 41 | 22 | 0.167 | 0.823 | 1.3 | 7.5 | 877.4 | 0.169 |
| 38 | 23 | 0.183 | 0.899 | 1.2 | 10.2 | 871.4 | 0.181 |
| 61 | 23 | 0.108 | 0.947 | 1.1 | 4.2 | 876.3 | 0.174 |
| 71 | 23 | 0.080 | 0.975 | 1.0 | 3.2 | 872.0 | 0.129 |
| 29 | 24 | 0.297 | 0.517 | 2.3 | 55.3 | 831.3 | 0.146 |
| 51 | 24 | 0.128 | 0.796 | 1.3 | 48.3 | 844.1 | 0.102 |
| 22 | 25 | 0.416 | 0.371 | 3.8 | 34.5 | 836.7 | 0.182 |
| 65 | 25 | 0.088 | 0.924 | 1.1 | 7.2 | 861.0 | 0.098 |
| 70 | 25 | 0.080 | 1.009 | 1.0 | 4.5 | 864.8 | 0.112 |
| 5 | 26 | 0.723 | 0.121 | 9.5 | 178.5 | 901.0 | 0.153 |
| 7 | 26 | 0.682 | 0.105 | 10.2 | 478.9 | 864.1 | 0.134 |
| 24 | 26 | 0.392 | 0.266 | 4.6 | 146.3 | 821.5 | 0.114 |
| 1 | 27 | 0.791 | 0.119 | 7.7 | 193.4 | 910.5 | 0.242 |
| 3 | 27 | 0.760 | 0.165 | 6.5 | 65.6 | 939.5 | 0.252 |
| 8 | 27 | 0.663 | 0.126 | 8.7 | 218.2 | 902.6 | 0.174 |
| 2 | 28 | 0.788 | 0.057 | 11.8 | 417.7 | 910.5 | 0.131 |
| 27 | 28 | 0.316 | 0.258 | 4.5 | 497.7 | 820.5 | 0.073 |
| 44 | 29 | 0.156 | 0.617 | 1.7 | 44.5 | 846.5 | 0.132 |
| 48 | 29 | 0.135 | 0.643 | 1.7 | 39.9 | 813.1 | 0.110 |
| 34 | 31 | 0.205 | 0.574 | 1.9 | 40.1 | 827.9 | 0.141 |
| 67 | 32 | 0.087 | 0.813 | 1.2 | 5.3 | 837.5 | 0.120 |
| 25 | 34 | 0.200 | 0.603 | 1.7 | 9.6 | 834.3 | 0.110 |
| 19 | 35 | 0.491 | 0.368 | 3.8 | 59.8 | 843.5 | 0.174 |
| 55 | 36 | 0.118 | 0.912 | 1.2 | 15.7 | 850.9 | 0.126 |
| 46 | 37 | 0.146 | 0.461 | 2.3 | 102.8 | 804.9 | 0.085 |
| 40 | 38 | 0.172 | 0.955 | 1.2 | 43.1 | 808.6 | 0.154 |
| 31 | 39 | 0.282 | 0.323 | 3.5 | 110.1 | 808.5 | 0.122 |
| 59 | 40 | 0.110 | 0.841 | 1.2 | 6.4 | 881.9 | 0.157 |
| 75 | 40 | 0.073 | 1.017 | 1.0 | 2.9 | 885.9 | 0.135 |
| 32 | 41 | 0.240 | 0.458 | 2.5 | 106.3 | 785.2 | 0.113 |
| 9 | 42 | 0.622 | 0.093 | 10.4 | 280.8 | 891.6 | 0.109 |
| 12 | 42 | 0.600 | 0.097 | 10.5 | 302.9 | 869.4 | 0.106 |
| 76 | 42 | 0.071 | 0.941 | 1.0 | 4.0 | 856.5 | 0.110 |
| 18 | 44 | 0.500 | 0.319 | 4.3 | 74.5 | 856.1 | 0.182 |
| 25 | 45 | 0.380 | 0.157 | 6.8 | 332.6 | 862.3 | 0.106 |
| 37 | 45 | 0.189 | 0.334 | 3.2 | 109.5 | 812.6 | 0.093 |

**Table S4: micro-CT values of age matched control samples.**

**Table S5: quantitative micro-CT scan evaluation of different PCBM phenotypes.**

**Table S6: evaluation of different samples obtained from individual patients.**

**Table S7: quantitative back-scattered scanning electron microscopy (qBSE-SEM) data of analyzed samples.**

|  |  | Trabecular bone | | Sclerotic bone | |
| --- | --- | --- | --- | --- | --- |
| Sample | Classification | Mean | St. Dev. | Mean | St. Dev. |
| 13 | Os WoT | 23.26 | 0.54 | 24.56 | 0.23 |
| 16 | Os Tra | 23.29 | 0.46 | 21.24 | 0.76 |
| 21 | Os Tra | 23.19 | 0.53 | 23.98 | 0.90 |
| 23 | Os WoT | 24.05 | 0.43 | 24.47 | 0.36 |
| 26 | Os Tra | 23.51 | 1.13 | 24.25 | 0.62 |
| 28 | Os WoT | 23.35 | 0.47 | 23.59 | 2.38 |
| 32 | Mixed | 23.67 | 1.64 | 21.63 | 1.12 |
| 35 | Mixed | 23.16 | 0.31 |  |  |
| 49 | Mixed | 23.33 | 0.44 | 23.51 | 0.59 |
| 52 | Mixed | 22.61 | 1.75 | 22.33 | 0.51 |
| 54 | Not affected | 24.02 | 0.65 |  |  |
| 68 | Lytic | 21.79 | 0.28 |  |  |
| 69 | Lytic | 22.97 | 0.44 |  |  |

Values in Ca weight %. Mean and St. Dev. of a minimum of 5 and up to 9 measurements per bone type in each sample.

**SUPPLEMENTAL FIGURES**


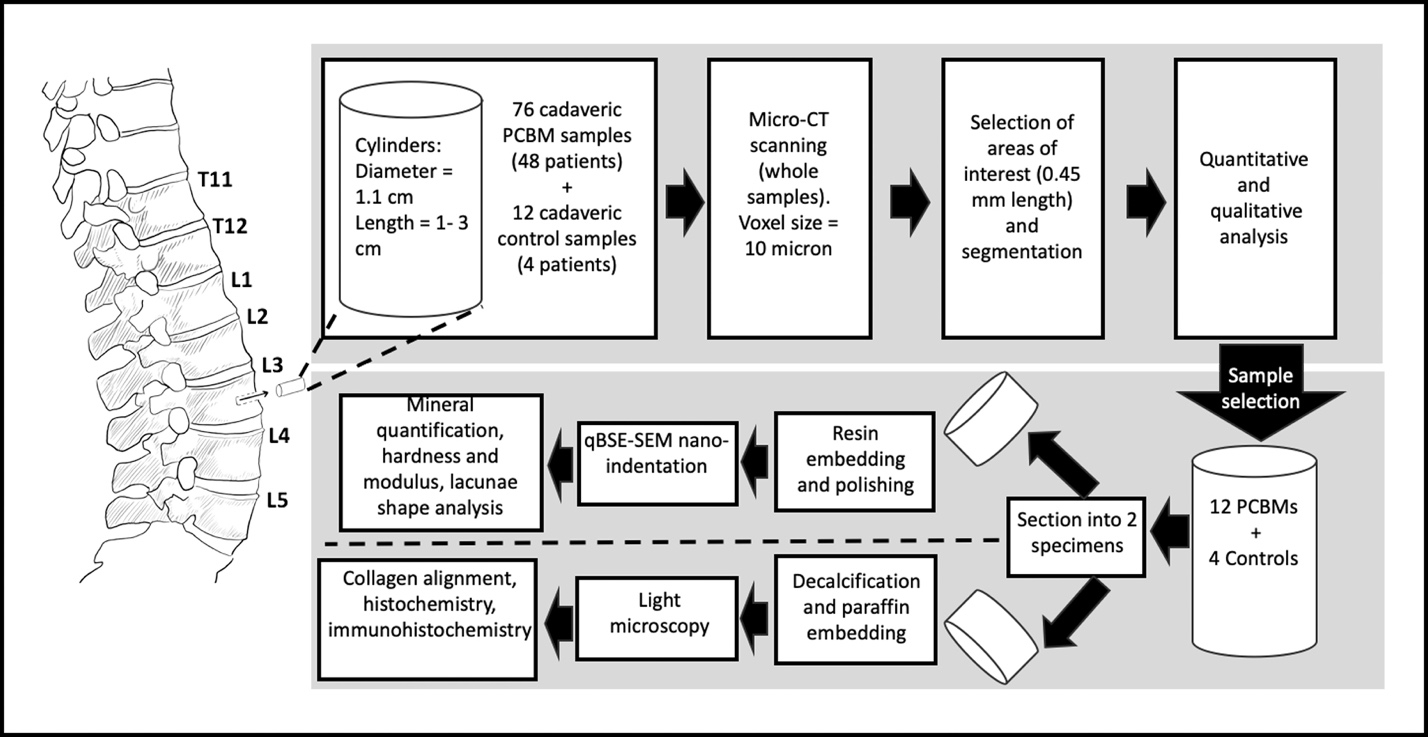


**Figure S1: Workflow of the samples and methods.**


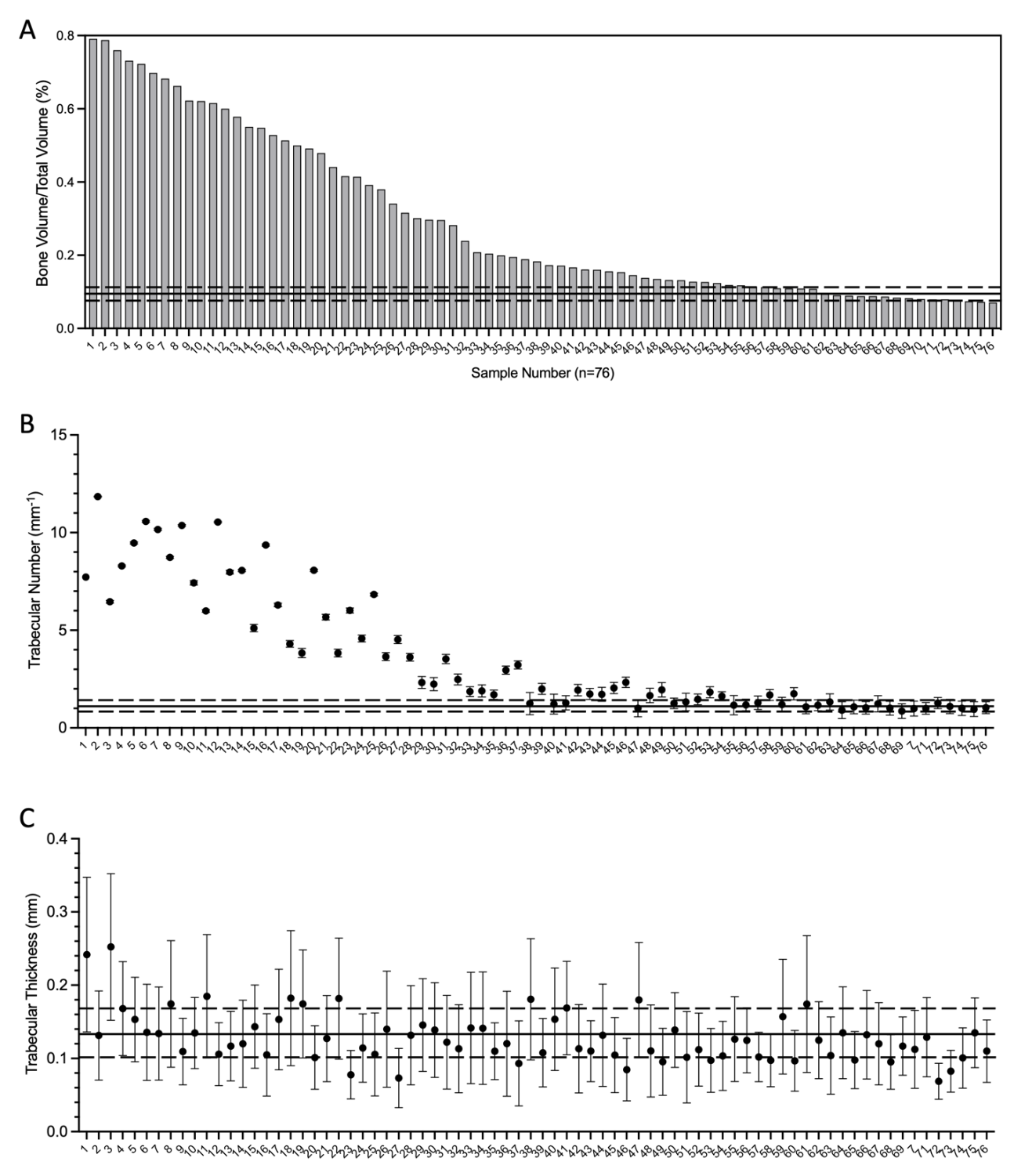


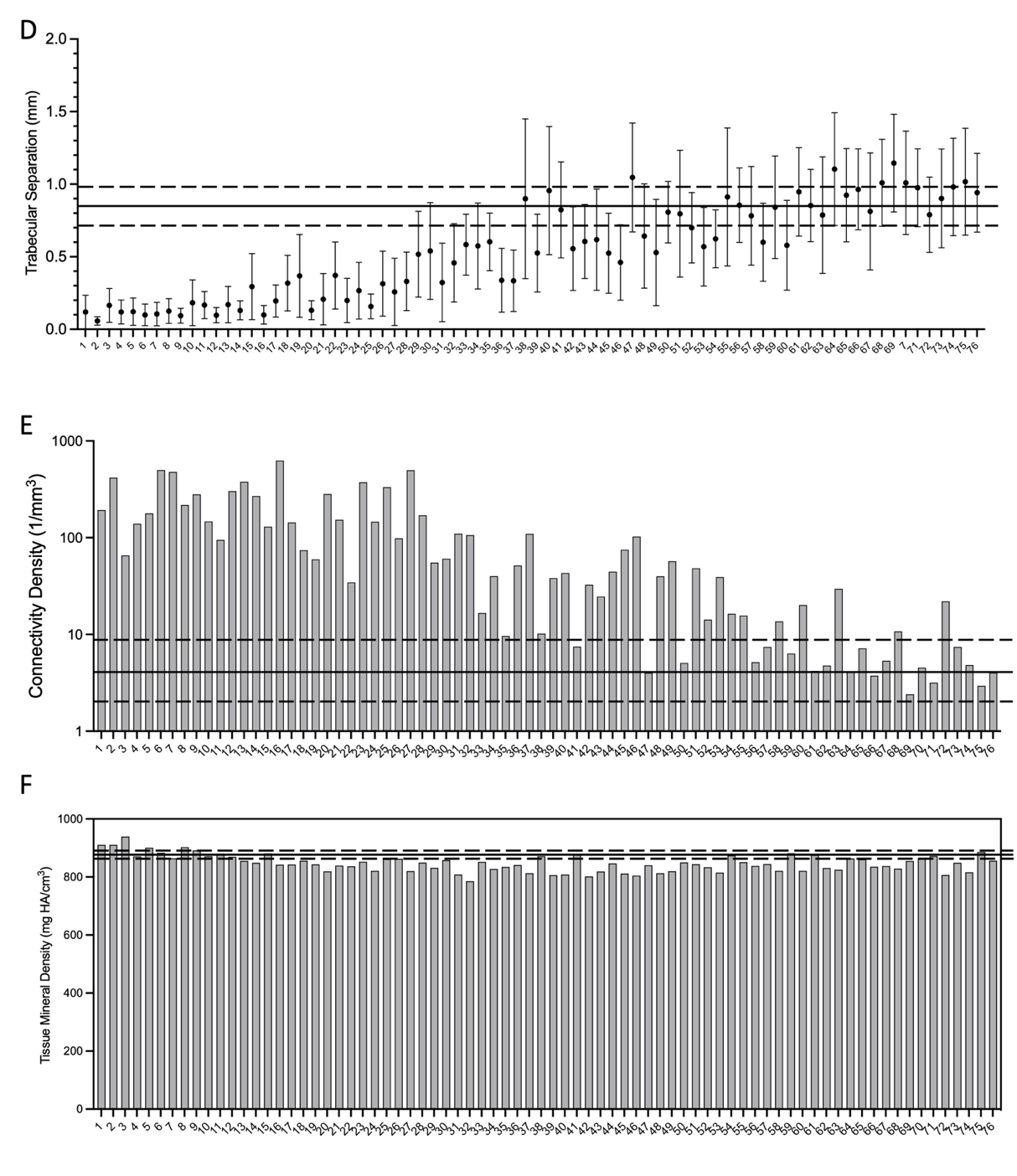


**Figure S2: Micro-CT analysis of cadaveric vertebral prostate cancer bone metastasis (n=76).** A) Waterfall plot ranking by bone volume over total volume of PCBM samples. B) Trabecular number by sq mm, mean value and SEM of each sample is shown. C) Trabecular thickness in mm, mean +- standard deviation is shown. D) intertrabecular space in mm, mean +- standard deviation is shown. E) Mean trabeculae connectivity density (number of connections per trabeculae) in PCBM samples (Chart in Log scale). F) Mineral density of the bone fraction (excluding medullary spaces) of PCBM samples, expressed in mg of hydroxyapatite/ cubic mm. Solid line, mean value, and dashed lines +- St. dev. of 12 age matched vertebral control samples.


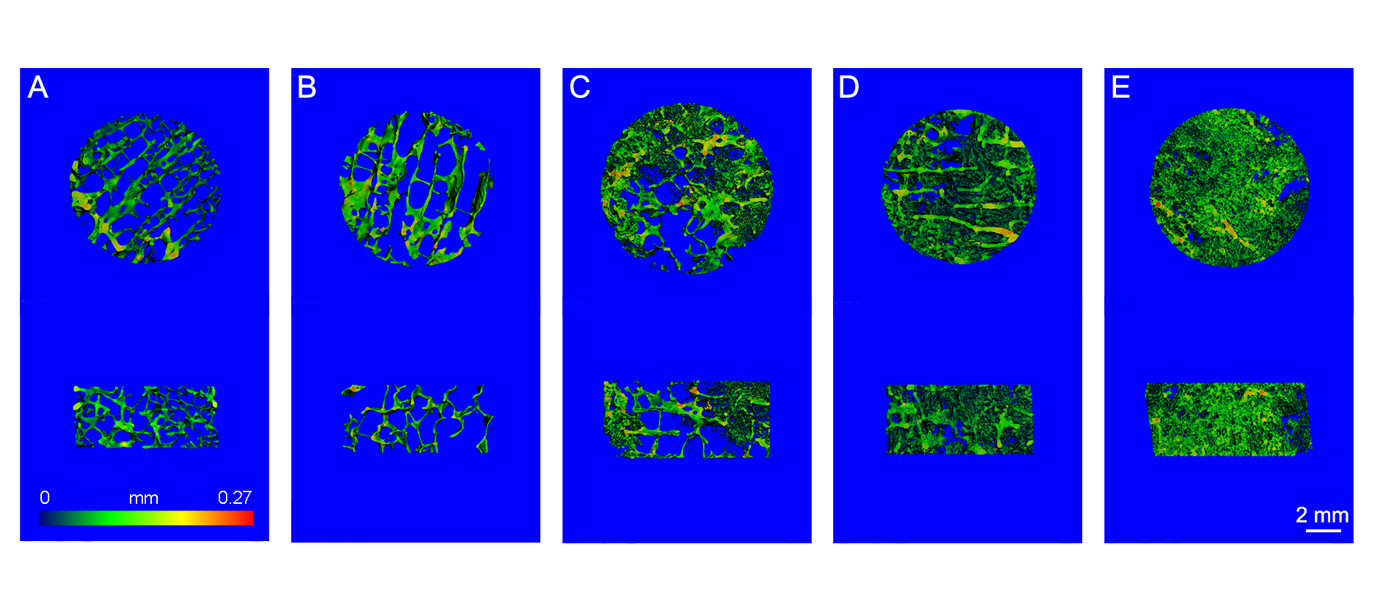


**Figure S3: Color-coded renders of representative cadaveric vertebrae of sub-types obtained from Micro-CT images (same samples and areas shown in Fig 2 in main text).** A) cadaveric age-matched control, B) osteolytic sample, C) Osteoblastic PCBM sample. E) Osteoblastic sample without residual trabeculae. Top images represent a transverse view and bottoms images are sagittal views of 1 mm thick sections. Renders were generated by superimposition of 6-micron sections. Color code represent trabecular thickness in the z axis.


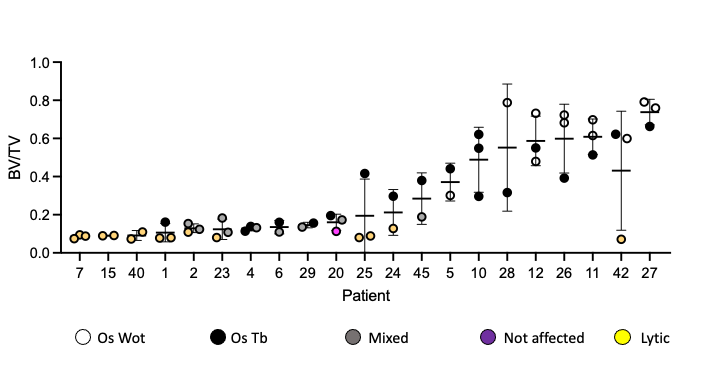


**Figure S4: BV/TV of samples grouped by patient.** The patients number corresponds with the numeration assigned in table S1. Color code: Yellow = Lytic, grey Mixed, Black= Os Tb, white= Os WoT.

**Fig S5: Waterfall plot of BV/TV of PCBM samples.** Red arrows indicate which samples were selected to perform SEM observation, and mineral composition analysis through qBSE.


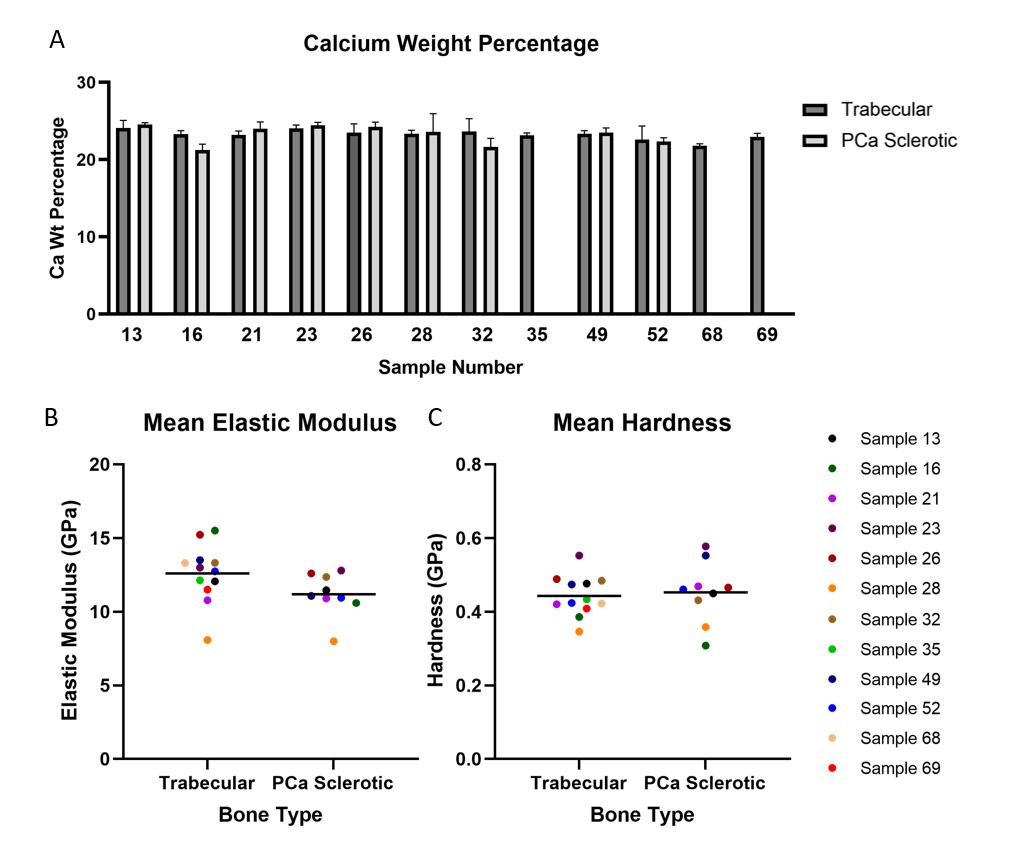


**Figure S6: Mineral content and nanomechanical properties of PCBM.** A) Calcium weight percentage of cadaveric vertebral prostate cancer bone metastasis samples measured by quantitative backscattered electron SEM. The mean value and standard deviation of the median Calcium weight percentage of each image is shown (images per sample per bone type = 5-9). B) Elastic Modulus and C) Hardness measurements for cadaveric vertebral prostate cancer metastasis samples (n=12). The mean value for each sample in trabecular regions (indents per sample=14-23) and sclerotic regions (indents per sample=7-23) are shown as individual dots. Black lines represent the mean values for all samples.

**Figure S7: Linear regression between hardness and modulus with Ca content:** Elastic modulus and hardness measurements for cadaveric vertebral prostate cancer metastasis samples (n=12, indents per sample=7-23) are plotted with the calcium weight percentage of the same area measured by quantitative backscattered electron SEM. Samples imaged = 13, 16, 21, 23, 26, 32, 35, 49, 52, 54, 68, 69.


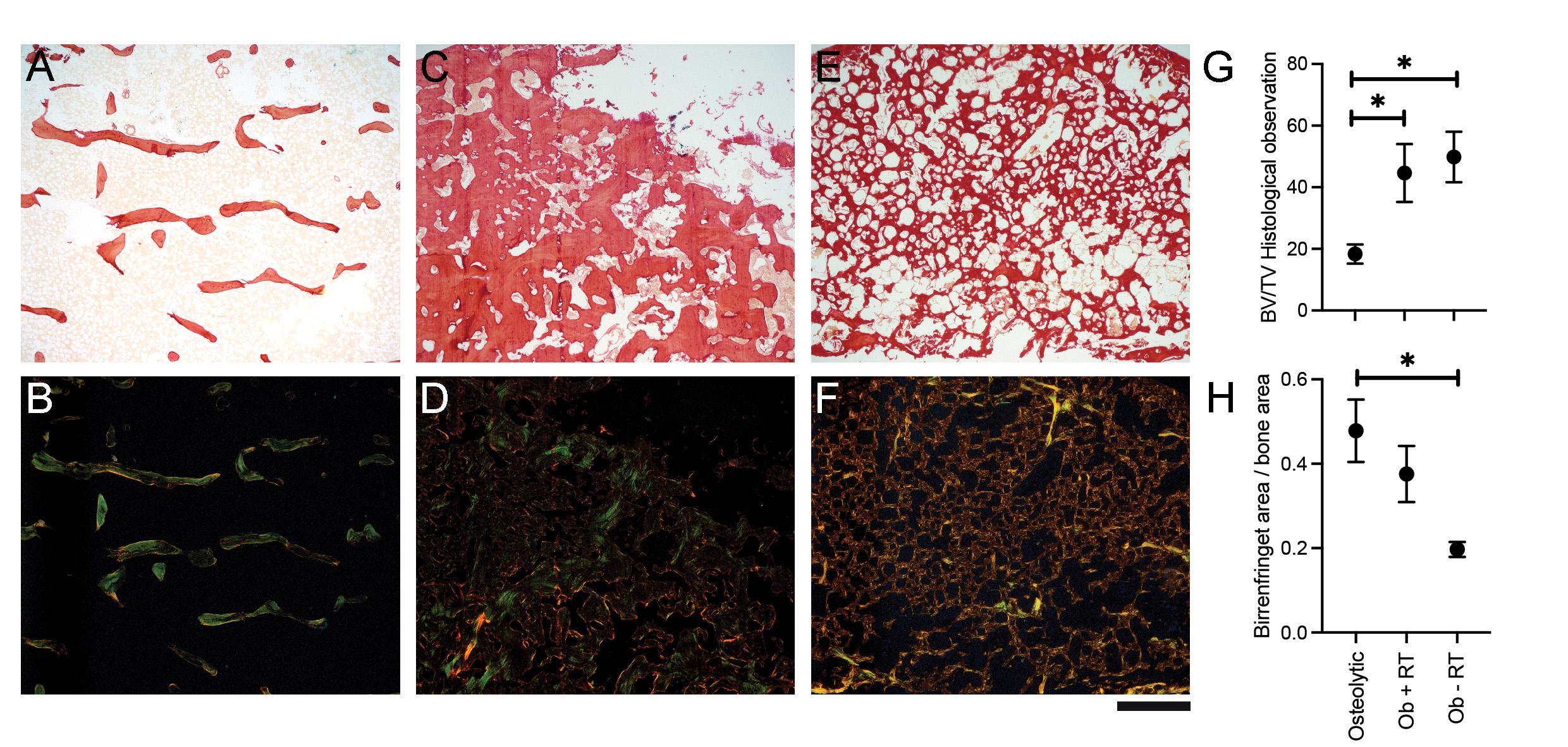


**Figure S8: Histomorphometry of PCBM.** A) Representative image of osteolytic PCBM specimen visualized with bright field. B) Polarized light imaging of A. C) Representative image of osteoblastic with residua trabeculae (OB + RT) PCBM specimen visualized in bright field. D) Polarized light imaging of C. E) Representative image of osteoblastic without residual trabeculae (OB - RT) PCBM visualized in bright field. F) Polarized light imaging of E. G) chart of mean +-- standard deviation of bone volume / total volume of different PCBM types. H) chart of birefringence area/ bone area of different PCBM types. * = p-value < 0.05. Staining= Picrosirius red. Scale bar = 1 mm. n = 6 osteolytic, 3 osteoblastic + residual trabeculae, 3 osteoblastic without residual trabeculae.

**
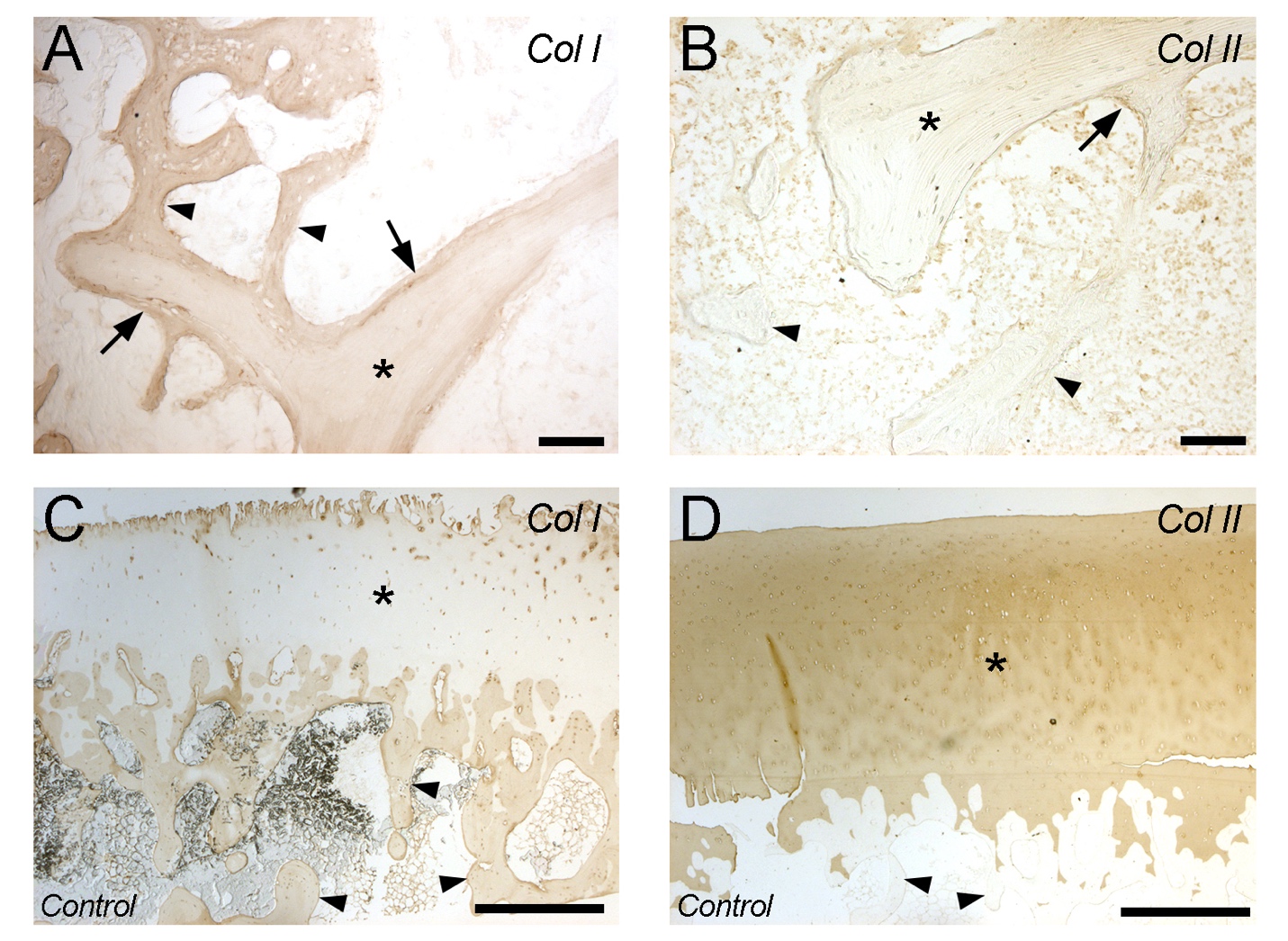
**

**Figure S9. Immunohistochemistry of collagenous matrix.** A) Immunodetection of Col I in PCBM shows staining of residual trabecular matrix (Asterisk), and a higher intensity in the sclerotic bone deposited in the trabecular surface (arrows) and in the intertrabecular spaces (arrowheads). B) Immunodetection of Col II resulted negative in all samples for both the residual trabecular matrix (asterisks), and the osteosclerotic bone in the trabecular surfaces (arrows) and the inter trabecular spaces (arrowheads). C and D) technical controls for Col I and Col II, articular cartilage (asterisks) is negative for Col I, but positive for Col II, while the underlying bone (arrowheads) stains positive for Col I but negative for Col II. Bar A and B = 100 microns, C and D = 250 microns.
